# A Multistep Computational Approach to Achieve a Complete Human 5‐Lipoxygenase Structure and Provide a Pharmacophore Model for Further Drug Design

**DOI:** 10.1002/minf.70025

**Published:** 2026-03-25

**Authors:** Lisa Lombardo, Francesco Agnello, Rosaria Gitto, Laura De Luca

**Affiliations:** ^1^ Department of Chemical, Biological, Pharmaceutical, and Environmental Sciences University of Messina Messina Italy

**Keywords:** full‐length human 5‐LOX, molecular docking, molecular dynamics, pharmacophores, protein model validation

## Abstract

Human 5‐lipoxygenase (5‐LOX) plays a crucial role in the biosynthesis of leukotrienes (LTs). Therefore, 5‐LOX inhibitors are designed as effective agents for the treatment of several diseases such as asthma, cardiovascular disorders, allergies, and cancer. Insights into crystal structures of several 5‐LOX isoforms have revealed that this protein adopts two different conformations (open/closed) through modulation of its Hα2 and arched helix regions, which are conditioned by the presence or absence of ligand in the active site; moreover, these structures are incomplete in regions critical for ligand binding. To advance the design of 5‐LOX inhibitors, we developed a computational procedure to reconstruct the first full‐length open conformation structure of 5‐LOX complexed with chelating inhibitor within the active site. Dynamic simulations and protein model validation confirmed the quality of our model, which was subsequently used for docking analyses and culminated in the development of a structure‐based pharmacophore model. These computational studies might constitute powerful tools for rationally designing and identifying novel 5‐LOX iron chelator inhibitors.

## Graphical abstract

Starting from incomplete structural information of 5‐lipoxygenase (5‐LOX), a multistep computational pipeline in silico led to powerful tools for the rational design and identification of novel chelator inhibitors. A reconstruction of the first full‐length 5‐LOX was performed and used to apply docking studies to enable to create a pharmacophore model.

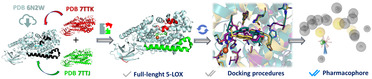

## 1 Introduction

**FIGURE 1:**
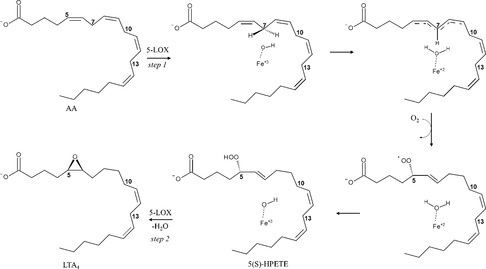
Schematic representation of the reaction mechanism catalyzed by 5‐LOX.

Lipoxygenases (LOXs, E.C. 1.13.11.34) are a family of nonheme iron‐containing dioxygenases widely distributed in plants and animals, they catalyze the regio‐ and stereospecific oxygenation of polyunsaturated fatty acids containing a *cis*, *cis*‐1,4‐pentadiene moiety. LOXs use arachidonic acid (AA, Figure 1) as the typical mammalian substrate and linoleic acid in plants [1, 2]. Different LOX isoforms (5‐LOX, 8‐LOX, 12‐LOX, and 15‐LOX) are classified based on the position where they insert molecular oxygen into the fatty acid backbone [3, 4, 5]. Human 5‐LOX is particularly relevant in physiopathological processes, as it synthesizes leukotrienes (LTs), potent pro‐inflammatory mediators involved in asthma, cardiovascular disorders, allergies, and various cancers [6, 7, 8].

5‐LOX catalyzes a two‐step conversion of AA into 5(*S*)‐hydroperoxyeicosatetraenoic acid (5(*S*)‐HPETE), which is then dehydrated to form the unstable epoxide LT A_4_ (LTA_4_, Figure 1)

Subsequently, LTA_4_ is converted to LTB_4_ by LTA_4_ hydrolase or to cysteinyl LT (LTC_4_) by LTC_4_ synthase [9, 10, 11]. In turn, LTC_4_ can be further converted into other cysteinyl LTs, such as LTD_4_ and LTE_4_.

**FIGURE 2:**
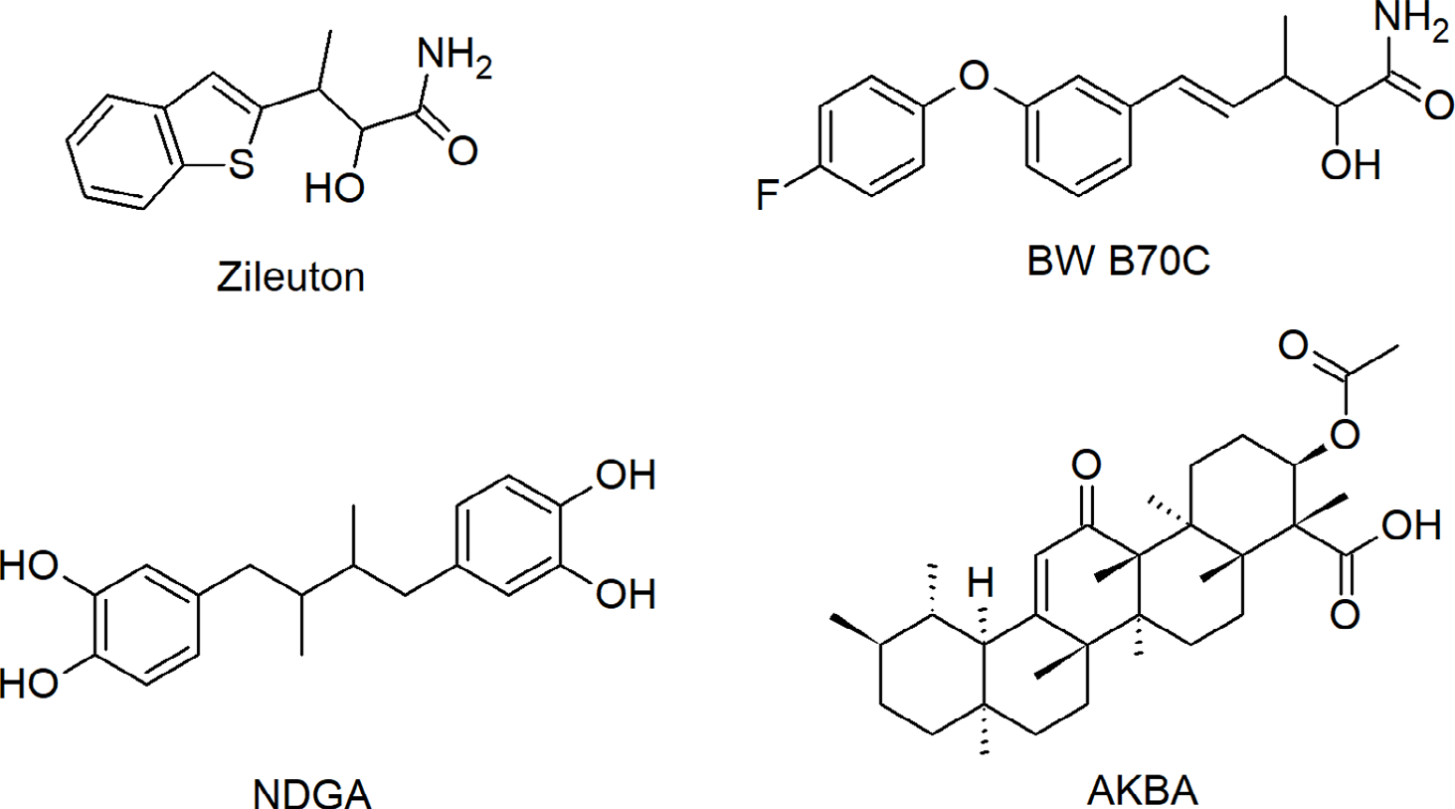
Chemical structures of well‐known iron‐chelating inhibitors zileuton, BW B70C, and NDGA, as well as allosteric inhibitor AKBA.

There are various therapeutics acting as 5‐LOX inhibitors through different modes of action, including competitive inhibitors such as zileuton, BW B70C, and NDGA, as well as the allosteric inhibitor acetyl‐11‐keto‐*β*‐boswellic acid (AKBA) (Figure 2).

Despite the clinical approval of zileuton (1‐[1‐(1‐benzothiophen‐2‐yl)ethyl]‐1‐hydroxyurea, Figure 2) several concerns regarding its toxicity profile emphasize the pressing need to develop novel, selective, and more effective inhibitors [12, 13, 14]. In this regard, different pharmaceutical companies and research groups are developing alternatives to zileuton.

Specifically, new agents are characterized by N‐hydroxyurea as a common pharmacophoric feature. In particular, BW B70C (1‐[(E)‐4‐[3‐(4‐fluorophenoxy)phenyl]but‐3‐en‐2‐yl]‐1‐hydroxyurea [15] (Figure 2) possesses the *N*‐hydroxyurea fragment, demonstrating high inhibitory effects (IC_50_ value of 0.08 μM) when compared to other known compounds [15]; BW B70C progressed into Phase III clinical study for knee osteoarthritis [13, 16].

The structure‐guided development of novel 5‐LOX inhibitors is currently hindered by the lack of complete 5‐LOX protein structures in complex with ligands available in the Protein Data Bank (PDB). Therefore, this study aims to computationally address this structural limitation by obtaining a full‐length structure suitable for guiding the structure‐based development of a comprehensive pharmacophore model for further design of 5‐LOX inhibitors.

## 2 Results and Discussion

### 2.1 Analysis of the Structure 5‐LOX

Human 5‐LOX possesses a conserved two‐domain architecture characteristic of the LOX family: a) an N‐terminal *β*‐barrel C2‐like domain mediating calcium‐dependent membrane association, and b) a larger C‐terminal *α*‐helical domain containing the active site with catalytic nonheme iron [17, 18] coordinated by crucial residues His367, His372, His550, Ile673, and Asn554 [7, 19, 20, 21, 22]. A detailed description of 5‐LOX structure is reported in Figure S1 of Supporting Information.

5‐LOX binding site is deeply located within the catalytic domain, and its accessibility is regulated by conformational changes of the two conserved helices, H*α*2 (171–197) and the arched helix (414–429). In detail, H*α*2 acts as a gate, modulating the open/closed conformation and controlling substrate access, with residues Phe177 and Tyr181 forming a “cork” that blocks the cavity. The arched helix encloses the binding site and contains an invariant Leu414 that is potentially critical for the substrate positioning [23].

In this study we carried out a comparative analysis of nine crystallographic structures of human 5‐LOX currently available [9, 23, 24, 25] in PDB (Table 1). All structures have been obtained by mutating fourteen residues to stabilize the enzyme for crystallization without affecting catalytic activity. These mutations improved the enzyme solubility and stability, making it more suitable for structural and functional studies [18]. The mutant enzyme, designated as Stable‐5‐LOX, was derived from a soluble 5‐LOX variant that presented the following modifications: a deletion of membrane insertion residues (Δ40 to 44GlySer), amino acid substitutions (Trp13Glu, Phe14Hys, Trp75Gly, and Leu76Ser), cysteine replacements (Cys240Ala and Cys561Ala), and substitution of a lysine‐rich region, LysLysLys with GluAsnLeu at positions 653–655.

**TABLE 1:** Human 5‐LOX PDB entries, resolution, presence of H*α*2 and arched helix, H*α*2 conformation, and eventual cocrystalized ligands.

PDB ID	Resolution (Å)	H*α*2 helix	Arched helix	H*α*2 helix conformation	Ligand
3O8Y [18]	2.39	Yes	Yes	Closed	No
3V92 [9]	2.74	Yes	Yes	Closed	No
3V98 [9]	2.07	Yes	Yes	Closed	No
7TTK [24]	1.98	Yes	Yes	Closed	No
7TTL [24]	2.43	Yes	Yes	Closed	No
6NCF [23]	2.87	Yes	Yes	Closed	AKBA
3V99 [9]	2.25	No	No	n.r.^a^	AA
6N2W [23]	2.71	No	No	n.r.^a^	NDGA
7TTJ [24]	2.10	Yes	No	Open	No

a n.r. = not resolved.

Data reported in Table 1 revealed that the H*α*2 helix adopted a closed conformation in five apo Stable‐5‐LOX structures (PDB IDs: 3O8Y, 3V92, 3V98, 7TTK, and 7TTL), thereby restricting the access to the catalytic site; these limitations hindered their use in the development of pharmacophore models. The binary complex with the allosteric inhibitor AKBA (see Figure 2; PDB ID: 6NCF) [23] also displayed a closed H*α*2 conformation, implying that the allosteric ligand binding did not trigger conformational changes for pocket opening. Stable‐5‐LOX has been crystallized both with its endogenous substrate AA (PDB ID: 3V99) and with the orthosteric inhibitor nordihydroguaiaretic acid (NDGA, see Figure 2; PDB ID: 6N2W). For the two structures 3V99 and 6N2W, the absence of an H*α*2 helix likely reflected conformational disorder induced by ligand occupancy of the active site. Notably, the only structure with a clearly resolved open conformation of the H*α*2 helix was the apo form 7TTJ.

Even if these nine structures have provided insights into molecular interactions and regulatory mechanisms governing 5‐LOX activity, the structural uncertainty of H*α*2 and the arched helix regions currently hampers the design of new inhibitors. Indeed, homology model strategies have been applied to gain structural and functional insights into 5‐LOX and overcome the issues arising from its conformational flexibility [26, 27, 28, 29].

In this context, the incomplete structural data for the 5‐LOX/NDGA complex in structure 6N2W have necessitated a computational approach to reconstruct the missing H*α*2 and arched helix regions; based on the results of studies performed by Gilbert et al., NDGA binding was considered inconsistent with a closed conformation, thus supporting the need for an open‐state model [30] Additionally, the open conformation has been associated with higher catalytic activity, as mutations stabilizing H*α*2 in this state increased reaction rates and substrate affinity. Although informative, these findings suggested that inhibitor‐induced conformational changes were distinct from those prompted by the native substrate [24]. As a consequence, the reconstruction of H*α*2 and the arched helix might enable a more accurate representation of pocket size and geometry to guide the computational studies. Finally, a preliminary comparison of the human 5‐LOX (PDB 6N2W) [23] with the physiologically relevant 12‐LOX (PDB 8GHB) [31] and 15‐LOX (PDB 4NRE) [32] isoforms suggested that the overall architecture of the LOX catalytic site was highly conserved. The description of the structural alignment was reported in Figure S2 of Supporting Information. Based on this analysis, we propose that our reconstructed protein could offer structural insights for structural elucidation of various LOXs, without providing pharmacophore insights on isoform selectivity.

### 2.2 Reconstruction of Full‐Length Stable‐5‐LOX–NDGA Complex

**FIGURE 3:**
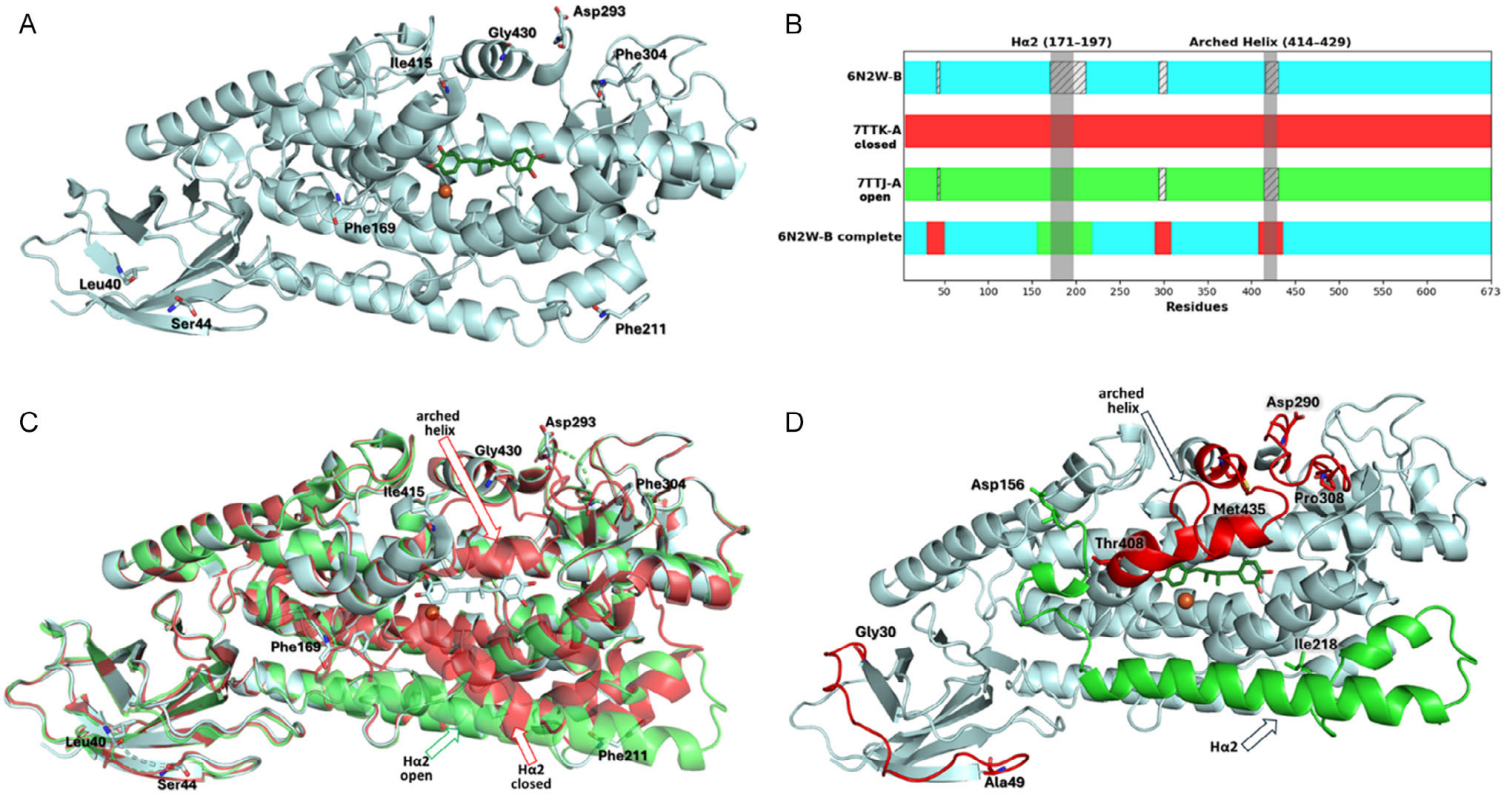
(A) Chain B of the crystal structure of 5‐LOX in complex with NDGA (PDB ID 6N2W). Residues at missing fragments are detected as sticks colored in pale cyan, while the remaining protein is reported as cartoon. The inhibitor NDGA is reported as a green stick, while the catalytic iron is reported as a brown sphere. (B) Missing loops in crystal structures 6N2W chain B (cyan), 7TTJ (green), and 7TTK (red) chain A and the loop insertion to obtain the complete structure (6N2W‐B complete). The dashed areas indicate missing residues, while the gray bands identify the H*α*2 (171–197) and Arched Helix (414–429) domains. (C) Superimposition of the 5‐LOX enzyme complexed with NDGA (PDB ID: 6N2W, in pale cyan) and its apo form in open (PDB ID: 7TTJ, in green) and closed (PDB ID: 7TTK, in red) conformations. (D) Reconstructed and minimized 5‐LOX; the residues used for attachment are indicated as sticks and labeled, whereas the H*α*2 chain taken from the PDB 7TTJ is colored as red. The arched helix and the two remaining loops taken from the PDB 7TTK are colored as green.

To reconstruct the full‐length stable 5‐LOX, we primarily focused on the stable‐5‐LOX/NDGA inhibitor complex (PDB ID 6N2W) [23]. This complex existed as a dimeric organization (chains A and B), bearing the ligand bound exclusively to chain B. The competitive binding induced significant structural rearrangements, leading to the formation of a deep ravine in the catalytic domain. As displayed in Figure 3A, the following four loop regions of chain B were unresolved: (1) residues 41–43; (2) residues 170–210 (including the H*α*2 helix 171–197); (3) residues 294–303; and (4) residues 416–429 (arched helix). To overcome the missing structural information, we planned the reconstruction of the complete 6N2W chain B/NDGA complex with the H*α*2 helix in an open conformation, as schematically illustrated in Figure 3B.

We decided to use the high‐resolution apo structures 7TTK (red) and 7TTJ (green) to provide a robust framework for modeling the missing segments in 6N2W chain B (cyan). Actually, the 7TTJ represents the only available crystal structure capturing an open conformation of the H*α*2 helix, which is required to accommodate orthosteric ligands, but it lacked several amino acids: (1) residues 41–43; (2) residues 294–301; (3) residues 416–429 (arched helix); whereas 7TTK presented a closed conformation of the H*α*2 helix but it was fully resolved, including the missing regions in 7TTJ.

It was worth noting that the 6N2W, 7TTJ, and 7TTK structures were resolved as homodimers. However, the functional 5‐LOX displayed a single polypeptide chain [33] as a feature critical for its biological activity. Therefore, we decided to focus our analyses on a single chain. In detail, chain B of 6N2W was selected as the template structure because it contained the NDGA in the catalytic pocket, while complete chain A was chosen for the 7TTJ and 7TTK structures.

Therefore, the crystal structures 7TTJ and 7TTK were superimposed onto structure 6N2W using PyMOL (Figure 3C). This structural alignment procedure produced highly similar conformations, exhibiting backbone root‐mean‐square deviation (RMSD) values of 0.258 Å and 0.273 Å for 7TTK and 7TTJ, respectively, when compared to 6N2W. The resulting aligned structures were then imported into Maestro to complete the final molecular assembly; missing regions corresponding to the H*α*2 helix, the arched helix, and additional loop segments were rebuilt using structural information from high‐resolution apo 5‐LOX structures. Loop integration was performed by selecting structurally compatible anchor points flanking each missing region, ensuring continuous backbone geometry and preserving the native secondary‐structure context of H*α*2 and the arched helix (see Materials and Methods section).

By preserving backbone continuity and native secondary‐structure context, the loop‐integration strategy allowed a coherent definition of the 5‐LOX binding pocket architecture, including its depth and steric limitations. This was essential for subsequent docking, molecular dynamic (MD) simulations, and pharmacophore development.

Thus, the desired complete 6N2W‐B structure was optimized using a minimization procedure as reported in the Materials and Methods section.

Briefly, the resulting model was subjected to structure preparation and energy minimization, followed by a localized refinement of the protein‐ligand complex to optimize active‐site geometry and ligand interactions.

This refinement step optimized local interactions and enhanced the quality of the protein–ligand complex, ensuring a better representation of chemical interactions and active site geometry. Through this multistep computational approach, we successfully reconstructed the full molecular architecture of the 5‐LOX/NDGA complex (Figure 3D). This model was considered a high‐quality structure based on validation through VERIFY 3D [34] and PROCHECK [35] via SAVES server (SAVES v6.1, Structure Validation Server (Los Angeles: UCLA‐DOE LAB, 2024), https://saves.mbi.ucla.edu/) (Figure S3 in Supporting Information).

To thoroughly examine protein–ligand complex stability, a 200 ns MD simulation was subsequently performed using the Desmond tool [36] from the Schrödinger Suite.

The stability of protein‐ligand complexes throughout the simulation was confirmed by analyzing the RMSD plot generated from the MD trajectories (Figure S4, Supporting Information).

This computational analysis was based solely on direct and persistent ligand‐residue contacts, without accounting for the water‐bridge interactions from the MD results. While this approach may not capture all aspects of the binding dynamics, we felt that the investigation of the persistent direct contacts was considered adequate for the purposes of our preliminary study.

**FIGURE 4:**
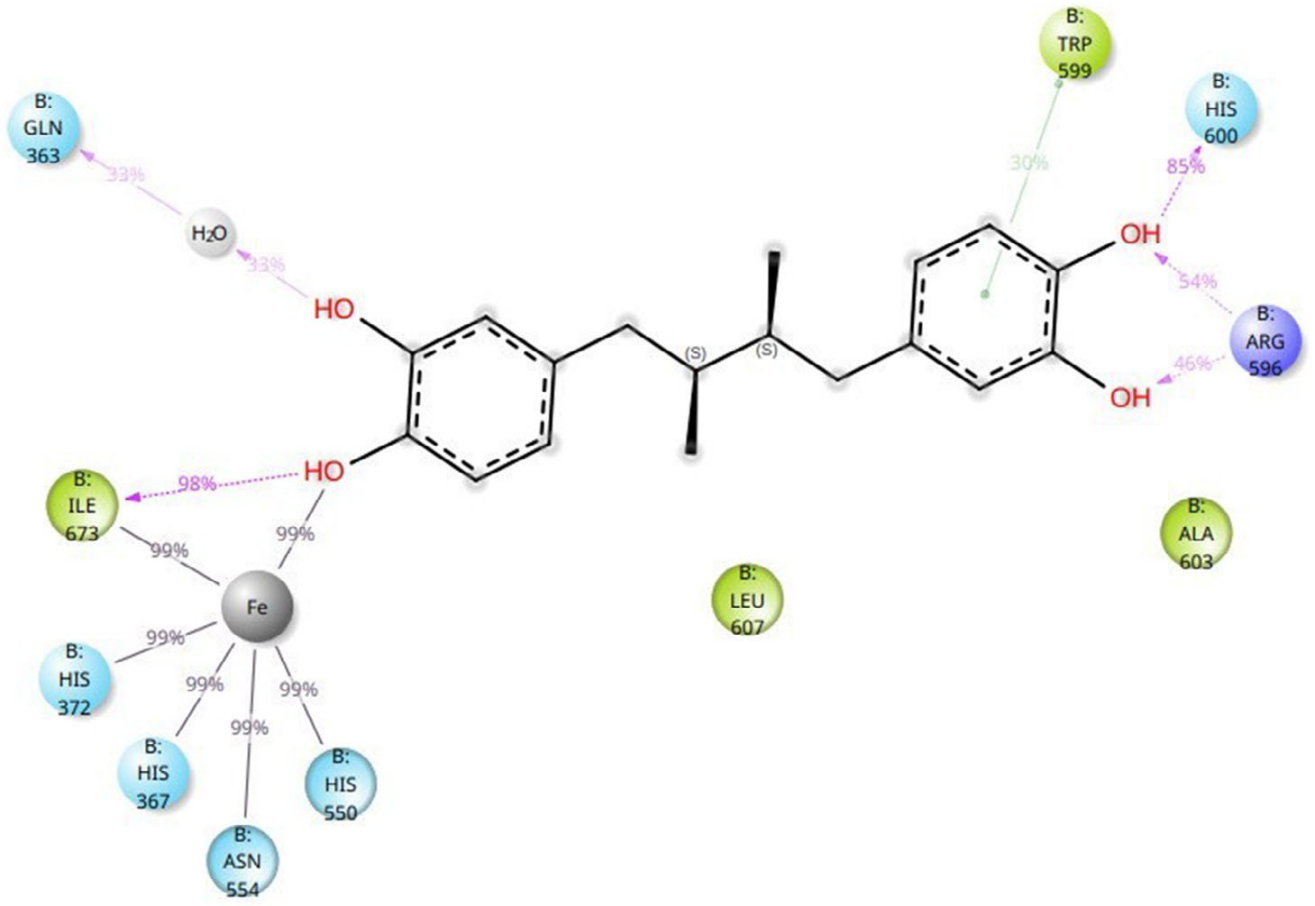
Detailed information about ligand functional groups/atoms that contact residues with a frequency higher than 25% of the simulation time.

Figure 4 displays the MD simulation results, highlighting key interactions between NDGA and 5‐LOX residues that occurred in more than 25% of the simulation time.

The most relevant contacts included: a) coordination of the catalytic iron by a hydroxyl group of the one catechol ring (99%), consistent with the mode of action of NDGA, that was categorized as redox and iron chelator inhibitor [13]; b) a highly stable hydrogen bond between this same hydroxyl and Ile673 (98%), stabilizing optimal ligand positioning; c) hydrogen bonding between the second catechol ring and His600 (85%).

The reconstructed 5‐LOX protein structure has been made available to support the identification of redox‐active and/or chelating 5‐LOX inhibitors at the following public link: https://github.com/lisa‐lombardo/5LOX‐full‐length‐structure‐and‐pharmacophore‐model.

### 2.3 Induced Fit Docking (IFD) Studies and Pharmacophore Model Development

**FIGURE 5:**
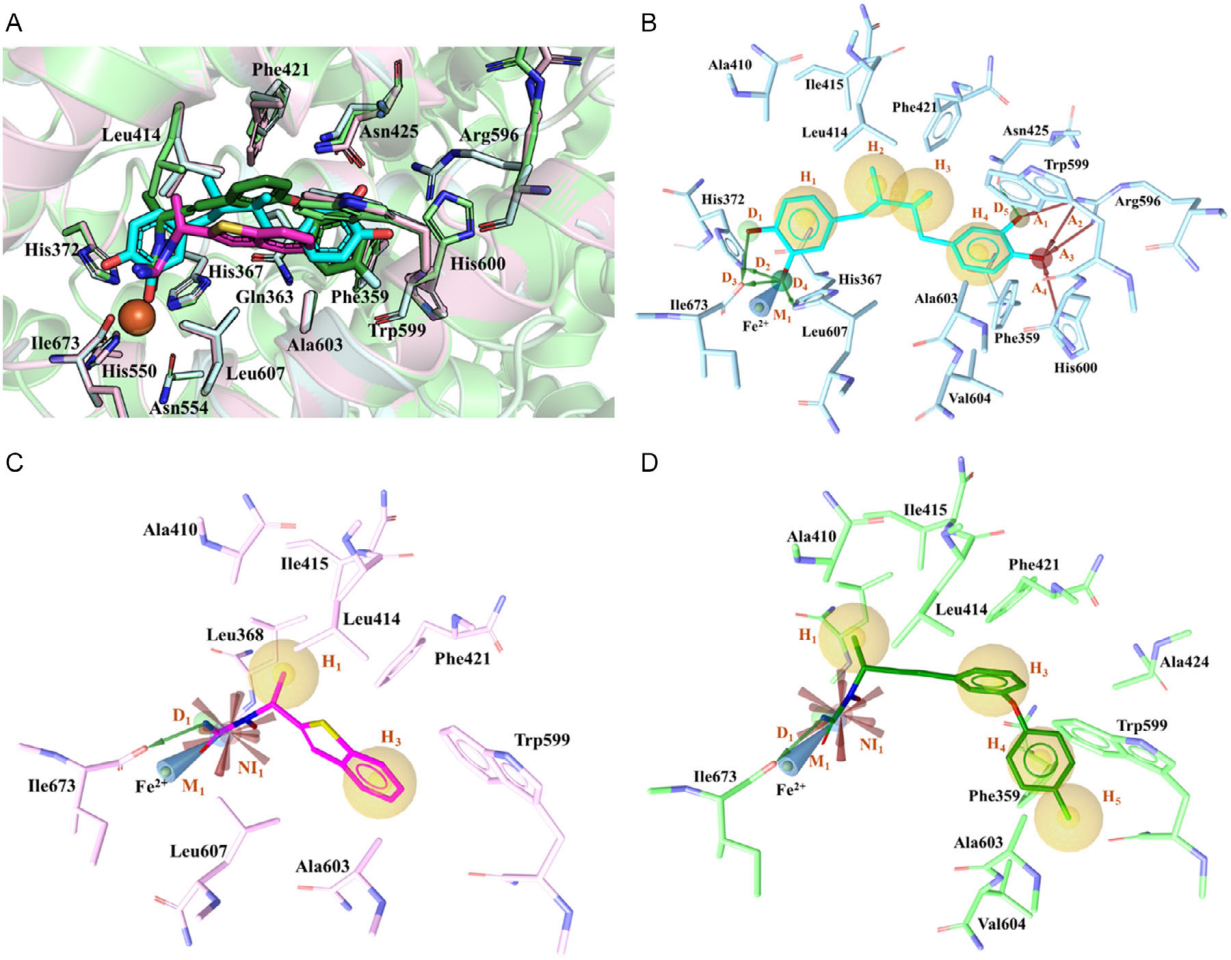
(A) Superimposition of IFD best poses of NDGA (cyan), zileuton (pink), and BW B70C (green) in the pocket of 5‐LOX. In the figure, interacting residues and ligands are represented as sticks, while the remaining protein is represented as cartoon. Fe ion is shown as a brown sphere, and hydrogen bonds are depicted as yellow dashes. The figure was created by Pymol (Schrödinger, L. & DeLano, W., 2020. PyMOL, available at: http://www.pymol.org/pymol). Pharmacophore models derived from the IFD complexes of (B) NDGA (cyan), (C) zileuton (pink), and (D) BW B70C (green). Residues and ligands are depicted as sticks, while iron ion as a sphere. Pharmacophoric features are represented as follows: hydrogen bond donors as green arrows, hydrogen bond acceptors as red arrows, hydrophobic regions as yellow spheres, iron‐binding location as a blue cone, and NI regions as stars. Excluded volumes are not shown for clarity.

After validating the 5‐LOX structure, IFD studies [37] were conducted to explore the binding modes of the iron chelators NDGA, zileuton, and BW B70C. IFD studies revealed that these compounds adopted a comparable binding orientation, primarily interacting with the catalytic site near the iron ion (Figure 5A). The information obtained from IFD was used to generate a structure‐based pharmacophore model for each 5‐LOX–ligand complex using LigandScout (v.4.5) [38]. In Figure 5B–D, there were displayed the pharmacophoric key features, including hydrogen bond donors (green arrows, D), hydrogen bond acceptors (red arrows, A), hydrophobic regions (yellow spheres, H), iron‐binding locations (blue cones, M), and NI regions (red stars, NI).

IFD studies highlighted that iron coordination and a hydrogen bond with Ile673 constituted the core interaction motif that was mediated by the catechol moiety in NDGA and the N‐hydroxy‐urea group in zileuton and BW B70C, thus confirming chelator inhibitory mechanism of the studied compounds. The functional relevance of Ile673 has been supported by mutagenesis studies showing that the alteration of this C‐terminal residue led to a marked loss of enzymatic activity [39]. Notably, these interactions were consistent with pharmacophore features M_1_ and D_1_, retained across all pharmacophore models, along with hydrophobic features H_1_ and H_3_, that represented the requirement for functional groups to establish favorable interactions with nonpolar residues located at the entrance of binding pocket (Leu368, Ala410, Leu414, Ile415, Leu607) and within the deeper cavity regions (Phe421, Trp599, Ala603). Remarkably, the reconstruction of missing regions, including the arched helix (residues 416–429), allowed us to define the complete ligand‐binding pocket and restore key interactions critical for inhibitor binding. In particular, the inclusion of residue Phe421, located within the arched helix, enabled the accurate modeling of hydrophobic contacts observed in all complexes that may contribute to ligand stabilization within the active site and would be otherwise lost in incomplete structures. Among the three ligands, NDGA exhibited the best interaction profile, featuring four additional hydrogen bond donors that were established with His372 (D_2_), Ile673 (D_3_), His367 (D_4_), and Asn425 (D_5_); four hydrogen bond acceptors were mediated by interactions with Arg596 (A_1_–A_3_) and His600 (A_4_); and two additional hydrophobic features engaged Phe421 (H_3_) and Phe359, Val604 (H_4_) (Figure 5B). Due to the smaller and rigid scaffold of prototype zileuton, it mainly relies on core M_1_ and D_1_ interactions and hydrophobic contacts, without fully engaging the deeper binding site. Its pharmacophore model included a NI feature (NI_1_) provided by the N‐hydroxy‐urea moiety (Figure 5C). In contrast, BW B70C, which is closer in size to NDGA, displayed intermediate complexity, incorporating an additional hydrophobic feature engaging Phe359 and Val604 (H_4_) like NDGA; moreover, there was a secondary hydrophobic interaction with Ala603 and Val604 (H_5_) and negative ionizable region (NI_1_) analogous to zileuton complex (Figure 5D).

The binding modes predicted by IFD studies were consistent with the known structure–activity relationships (SAR) of established 5‐LOX inhibitors. Iron chelation through catechol or N‐hydroxy‐urea moieties has been widely reported as a key determinant for 5‐LOX inhibition, as exemplified by NDGA and zileuton, whose activity critically depends on the ability to coordinate the catalytic iron ion [18, 40]. Previous SAR studies have shown that disruption or masking of the iron‐chelating group leads to a marked loss of activity, supporting the central role of metal coordination captured by the M_1_ and D_1_ pharmacophoric features [13, 41, 42].

In addition, hydrophobic substituents capable of engaging residues lining the substrate access channel and the deeper pocket region have been reported to enhance inhibitory potency in NDGA‐derived and related scaffolds, in agreement with the hydrophobic features (H_1_, H_3_, and H_4_) observed in the present docking and pharmacophore models [13]. The persistence of iron coordination and hydrophobic contacts observed during MD simulations further supports the relevance of these interactions, which are consistent with experimental SAR trends reported for iron‐chelating 5‐LOX inhibitors.

Interestingly, residue conformations within the binding site were largely conserved across complexes, with ligand‐specific adaptations observed for Arg596 and Leu414, thus highlighting the role of active site residues in adapting the pocket to ligand‐specific steric and interaction requirements.

Indeed, all pharmacophore models incorporated various excluded volumes representing sterically restricted regions that define the spatial limitations for ligand accommodation within the 5‐LOX active site.

To derive a consensus pharmacophore model for iron‐chelators 5‐LOX inhibitors, we applied a rational selection strategy based on: (i) identifying the most conserved features across all ligand‐target complexes and (ii) merging these features into a unified pharmacophore hypothesis. Beyond complex stability assessment, MD trajectory analysis was exploited to characterize binding‐site flexibility and interaction persistence. In particular, persistent interactions observed over the simulation time were distinguished from more transient contacts involving flexible residues such as Arg596. These observations were subsequently used to guide the prioritization and refinement of pharmacophoric features. Our analyses revealed that additional interactions with key residues were also crucial for this class of compounds. These included a hydrogen bond donor (D_1_) interacting with crucial Ile673, which likely facilitates coordination with the Fe^2+^ ion, and two hydrophobic moieties (H_1_ and H_3_) that engaged various contacts with nonpolar residues.

**FIGURE 6:**
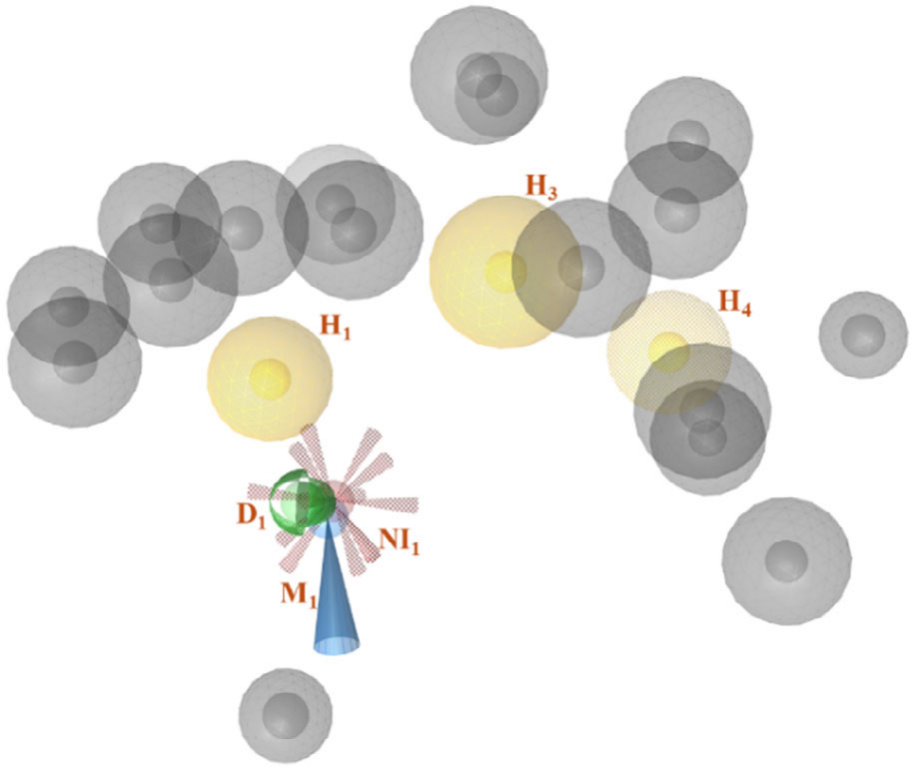
Final pharmacophore models gained by our consensus procedure. Pharmacophoric features are represented as follows: hydrogen bond donors as green arrows, hydrogen bond acceptors as red arrows, hydrophobic regions as yellow spheres, iron‐binding location as a blue cone, NI regions as stars, and excluded volumes as gray spheres.

Moreover, we chose to retain the hydrophobic feature H_4_, shared by NDGA and BW B70C, as well as the negative ionizable region (NI_1_), which was common to BW B70C and zileuton. Given the high variability that emerged in the deeper region of the pocket, features A_1_–A_4_ and H_5_ were excluded from the final model. Then, the excluded volumes were combined, retaining only the overlapping regions shared across all complexes. To broaden the applicability of the model, the D_1_ feature was converted from a directional vector to a nondirectional sphere. Features NI_1_ and H_4_ were set as optional, as they were observed in two out of the three complexes. The final consensus pharmacophore model consisted of six features: M_1_, D_1_, H_1_, NI_1_, H_3_, and H_4_, along with seventeen excluded volumes as displayed in Figure 6.

**FIGURE 7:**
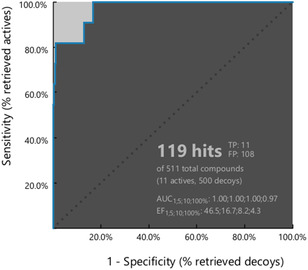
ROC curve generated by LigandScout, along with the corresponding AUC and EF output values.

In the last step of our study, we assessed the ability of the pharmacophore model to discriminate between active and inactive compounds; this analysis was carried out through a virtual screening procedure using a custom‐built validation set. The focused library consisted of eleven known active 5‐LOX inhibitors, including NDGA, BW B70C, zileuton, and other reported iron chelators retrieved from the literature (Table S1 in Supporting Information) [43, 44, 45, 46, 47, 48, 49, 50], alongside 500 decoy compounds. Given the limited availability of experimentally inactive molecules, the decoys were generated using the LUDe webapp [51] to simulate plausible inactive structures. Pharmacophore performance was evaluated using standard metrics implemented in LigandScout [38], including the receiver operating characteristic (ROC) curve, area under the ROC curve (AUC), and enrichment factor (EF) (Figure 7).

Notably, the model successfully identified all eleven active compounds (true positives) while misclassifying 108 decoys as active (false positives). This false‐positive rate was considered consistent with a pharmacophore model designed for early‐stage virtual screening and a scaffold discovery tool, where maximizing the selection of potential hits was prioritized.

The SMILES of the one hundred eight structures are shown in Table S2 in Supporting Information.

The resulting AUC value of 0.97 indicated excellent discriminatory power. The EF value of 4.3, calculated across the entire dataset, reflected a moderate enrichment capability, which should be interpreted in light of the limited number of active compounds available for validation. Such a limitation derived from the selection of inhibitors with well‐established iron‐chelating activity and reliable experimental evidence, chosen to ensure a high‐confidence validation set.

Notably, the pharmacophoric hypothesis successfully identified all known active inhibitors in the validation set, demonstrating high sensitivity and supporting its suitability as a hit‐finding tool. The relatively high number of false positives reflected the fact that the model was conceived for early‐stage virtual screening and scaffold discovery rather than strict selectivity filtering. In this context, prioritizing sensitivity and chemical space exploration was advantageous, as it reduced the risk of discarding potentially valuable chemotypes at the initial screening stage.

Selectivity can be progressively enhanced in downstream refinement steps through the application of complementary structure‐based approaches such as docking studies and MD simulations to evaluate interaction persistence and binding stability. Overall, these results supported the robustness and predictive capability of the proposed pharmacophore model, which should be confirmed by further experimental validation in future studies.

Although flexible loop regions such as H*α*2 and the arched helix were inherently challenging to resolve experimentally, their reconstruction proved to be essential for obtaining a complete and functionally meaningful representation of the 5‐LOX binding pocket.

Specifically, the reconstruction highlighted the role of Phe421 in contributing to hydrophobic interactions with inhibitors as well as the identification of sterically inaccessible regions in the final pharmacophore model.

For further research purposes in the identification of new redox‐active and/or chelating 5‐LOX inhibitors, the obtained pharmacophore model has been made available for support at the following public link: https://github.com/lisa‐lombardo/5LOX‐full‐length‐structure‐and‐pharmacophore‐model.

## 3 Conclusions

As the available structure of human 5‐LOX in complex with the competitive inhibitor NDGA contained missing regions critical for ligand binding, we tried to fill this gap through the in silico reconstruction of an open conformation of the full‐length and validated protein structure. The obtained final structure was submitted to a thorough quality assessment, including VERIFY 3D and PROCHECK analysis, confirming a high percentage of residues in favorable regions and overall compatibility between sequence and 3D conformation. MD simulations revealed the structural stability of the complex and key interactions.

Although our reconstruction was guided by experimentally resolved human 5‐LOX structures, we acknowledge that the obtained full‐length structure has not been clearly confirmed by direct experimental and external validation beyond internal geometric and MD‐based assessments. The lack of experimental validation might represent a limitation of the present study. To overcome this issue, the reliability of the reconstructed model was supported by the evaluation of its ability to reproduce experimentally reported binding modes and SAR of well‐characterized 5‐LOX inhibitors. While some degree of conformational uncertainty was intrinsic to flexible loop regions, the reconstructed 5‐LOX model enabled the definition of a complete binding pocket and supported the development of a pharmacophore model capturing key interaction features that were not accessible in incomplete experimental structures from PDB. IFD studies further elucidated the binding modes of well‐characterized iron‐chelating inhibitors, highlighting common pharmacophoric features essential for 5‐LOX inhibition. Finally, the derived structure‐based pharmacophore model demonstrated excellent discriminatory performance and a satisfactory EF, revealing robustness and potential use in virtual screening workflows. Overall, this work provides a reliable computational framework to further rationally design and identify novel 5‐LOX inhibitors able to bind the orthosteric site. In particular, the developed 5‐LOX structure and its derived pharmacophore model might offer a suitable platform for the research community that could be exploited in future fragment‐based virtual screening campaigns to expand chemical diversity and identify novel scaffolds beyond known iron‐chelating inhibitors.

## 4 Materials and Methods

### 4.1 Protein Preparation and Refinement

The align function of PyMOL (Schrödinger, L. & DeLano, W., 2020. PyMOL, Available at: http://www.pymol.org/pymol) was used to align chain A of 7TTJ and 7TTK [24] to chain B of 6N2W [30]. To build the missing loops in the protein structure, the Build function of the Maestro software, part of the Schrödinger Suite (Schrödinger Release 2021‐4: Maestro, Schrödinger, LLC, New York, NY, 2021) was used.

Once the structure was rebuilt, the optimization strategy was performed as follows:

a. Loop Integration Strategy: Instead of inserting the loops directly at truncation points, they were added both before and after the missing regions, by aligning with areas where the backbone and side chains of aligned proteins showed higher overlap. This methodology efficiently reconstructed the missing loops in 6N2W spanning four distinct regions: residues 30–49, 156–218, 290–308, and 408–435, using structural information from 7TTK and 7TTJ.
b. Structure Preparation: The reconstructed structure underwent comprehensive preparation using the Protein Preparation Wizard [52] which involved correcting errors in the structural model, properly assigning protons, and optimizing side chain geometry.
c. Local Minimization: Residues 414–430 were subjected to targeted minimization using the Prime tool [53] to resolve significant steric clashes characterizing this region.
d. Global Minimization: The entire protein, including iron and ligand components, was subjected to minimization with Cartesian constraints applied to the complete structure.
e. Final Refinement: The refinement was performed using the Refine Protein–Ligand Complex module [53, 54] (Schrödinger Release 2021‐4: Maestro, Schrödinger, LLC, New York, NY, 2021), focusing on the ligand and residues within a 5 Å radius of the ligand, except for residue Arg596 and certain residues of the arched helix. This decision was based on the observation that further minimization of Arg596 caused it to shift away from the binding pocket and to preserve the *α*‐helix formed by residues 420–425, which was reported as an ordered secondary structure in other crystal structures.

Protein preparation was performed using the Protein Preparation Wizard [52] from the Schrödinger Suite, with default parameters except for minimization, which was omitted.

For energy minimization of the structure, the Minimize module of Prime [53] was employed. The first minimization was performed on residues 414–430, with no constraints applied. The Quasi‐Newton (LBFGS) method was used with 5 iterations of 100 steps each and a convergence criterion of 0.01 kcal/mol. The OPLS4 force field was applied. The second minimization was performed on the entire protein, including the ligand and iron. Cartesian constraints were applied to the entire protein with a force constant of 5 kcal/(mol·Å^2^) and a tolerance distance of 0.2 Å. The automatic method was used for minimization with 4 iterations of 200 steps and a convergence criterion of 0.01 kcal/mol. The OPLS4 force field was applied. For the refinement of the protein–ligand complex, the ligand and residues within a 5 Å radius from the ligand (177, 359, 360, 367, 368, 372, 557, 569, 599, 600, 603, 604, 607, 673), excluding residue 596, were selected, along with residues 406–428. Local optimization was used as the refinement method, and the OPLS4 force field was applied.

### 4.2 Molecular Dynamic Simulation

The MD simulation of the reconstructed structure was performed using the Desmond tool [36] from the Schrödinger Suite. Initially, the complex was placed in a simulation box using the System Builder module, applying the default parameters. To mimic the effects of an aqueous environment, the TIP4P water model was selected as the explicit solvation model. An orthorhombic box was generated with a 10 Å buffer in all three dimensions. An NaCl saline solution at a concentration of 0.15 M was added to the system, and neutralization was achieved by adding Na^+^ ions. The “Minimize Volume” option was selected, and the OPLS4 force field was applied. The prepared system was subjected to MD simulation for a total duration of 200 ns, with trajectory frames recorded every 200 ps, resulting in 1000 frames. The simulation was carried out in the NPT ensemble (constant number of particles, pressure, and temperature), using a Nosé–Hoover thermostat and barostat to control thermodynamic conditions. Temperature and pressure were maintained at 300 K and 1 atm, respectively. The default relaxation times were applied: 1.0 ps for the thermostat and 2.0 ps for the barostat. The integration time step was set to the default value of 2.0 fs. Short‐range Coulombic interactions were treated using a short‐range method with a cutoff distance of 9 Å. The “Relax model system before simulation” option was enabled, which applies a series of energy minimizations and short MD simulations under NPT conditions to equilibrate the system prior to the production run.

### 4.3 Molecular Docking

Molecular docking was applied using the Induced Fit Docking tool [37] of the Schrödinger Suite. For each compound, up to 32 protomer/tautomer states were generated at pH 7.4 ± 1.0 using Epik with the OPLS4 force field. Stereochemistry was retained, and low‐energy ring conformers were enumerated. A docking box of 16 Å in size was defined, centered on the centroid of the ligand, with no constraints applied. Standard‐precision (SP) Glide was used for the initial placement (top 20 poses, van der Waals scaling = 0.5), and the complexes were rescored with Glide extra‐precision (XP). The best‐ranked pose for each protomer/tautomer was retained; the three highest‐scoring unique poses per ligand were exported for pharmacophore modeling. Within the advanced IFD settings, ring conformations were sampled by enabling the “Sample ring conformations” option and setting an energy window of 2.5 kcal/mol. For amide bonds, the “Penalize nonplanar conformation” option was activated. Regarding binding site flexibility, receptor residues within 5.0 Å of the ligand poses were refined by Prime module [53].

### 4.4 Pharmacophore Model Generation and Validation

The three optimal poses of each inhibitor (NDGA, zileuton, BW B70C) were imported into LigandScout (v4.5) [38]. Individual structure‐based pharmacophore models were generated, then the three models were aligned to using the NDGA pharmacophore as reference. A manual curation was carried out, specifically, redundant or chemically implausible features were deleted or merged; feature types were adjusted to match the hydrogen‐bond pattern observed in the crystal structure. The curated feature sets were merged into a single consensus model, and spatial tolerances were harmonized by the ‘Interpolate all features within tolerance’ function. Finally, overlapping or noninformative excluded volumes were removed to avoid over‐restriction of the binding pocket. The final model was evaluated for its effectiveness by screening a validation set. The dataset of active compounds was retrieved from the literature, with a careful selection of 11 compounds reported as 5‐LOX iron chelators [43, 44, 45, 46, 47, 48, 49, 50]: Baicalein, Atreleuton, BW A4C, BW A137C, BW B70C, CAPE, CHEMBL119499, CHEMBL421423, Honokiol, and zileuton. Additionally, 500 property‐matched decoys were generated using LUDe (LIDEB's Useful Decoys) [51], ensuring balance in molecular weight, logP, hydrogen bond donors/acceptors, and net charge. To evaluate the pharmacophore model, active and decoy compound libraries were generated using the ‘iCon Best Generation’ procedure available in LigandScout. Virtual screening was carried out with the following settings: the scoring function was set to ‘Pharmacophore‐Fit Score,’ the screening mode to ‘Match All Query Features,’ and the retrieval mode to ‘Get Best Matching Conformation’. No features were omitted, and the ‘Check Excluded Volumes’ option was enabled.

## Supporting Information

Additional supporting information can be found online in the Supporting Information section. **Supporting Fig. S1:** Schematic representation of the human 5‐LOX protein structure. The N‐terminal C2‐like domain (orange) is involved in calcium and membrane binding, while the C‐terminal catalytic domain (purple) contains the iron‐binding site. The key iron‐chelating residues His367, His372, His550, Asn554, and the terminal carboxyl group of Ile673 are located within the catalytic domain. **Supporting Fig. S2:** Structural superimposition of human 5‐LOX (PDB ID: 6N2W, pale cyan) [1] with human 15‐LOX‐2 (PDB ID: 4NRE, dark pink)[2] and human 12‐LOX (PDB ID: 8GHB, pale green) [3]. Residues lining the binding pocket are shown as sticks, while the catalytic iron ion is depicted as spheres. The remaining protein regions are represented as cartoons. **Supporting Fig. S3** **:** A) Graph resulted from VERIFY 3D analysis. The x‐axis indicates the number of residuals and the y‐axis the assigned score. We highlight the threshold set by the software of 0.1 B) Ramachandran plot obtained as an output of PROCHECK. In the diagram, the X‐axis represents the φ angle, while the Y‐axis represents the ψ angle. The red areas represent the favorable regions, the yellow areas the permitted regions, and the white areas the unfavorable regions. **Supporting Fig. S4** **:** RMSD chart of the ligand‐protein complex during molecular dynamic simulation. The x‐axis shows the simulation time, while the y‐axis shows the change in RMSD value of the ligand and protein during the trajectory. **Supporting** **Table** **S1:** List of compounds included in the active set for pharmacophore validation, along with their 2D chemical structures, reported IC_50_ values, and corresponding literature references. **Supporting** **Table** **S2** **:** List of decoys comprised in the inactive set for pharmacophore validation, along with their SMILE strings.

## Funding

This work was supported by the Ministero dell’Istruzione, dell’Università e della Ricerca (Grant PRIN 201744BN5T).

## Conflicts of Interest

The authors declare no conflicts of interest.

## Supporting information

Supplementary Material

## Data Availability

The reconstructed 5‐LOX protein structure and the pharmacophore model files are accessible via online repository https://github.com/lisa‐lombardo/5LOX‐full‐length‐structure‐and‐pharmacophore‐model).
