# Supplementary material for: A Multistep Computational Approach to Achieve a Complete Human 5‐Lipoxygenase Structure and Provide a Pharmacophore Model for Further Drug Design
Source: Mol Inform. 2026 Mar 25;45(3):e70025. doi: 10.1002/minf.70025 (PMC13014066; doi:10.1002/minf.70025)
Supplement: Supplementary file 1 — Supplementary Material [file MINF-45-e70025-s001.pdf]

Supporting Information

A multi-step computational approach to achieve a complete human 5-Lipoxygenase structure and provide a pharmacophore model for further drug design

Lisa Lombardo, Francesco Agnello, Rosaria Gitto, Laura De Luca*

Department of Chemical, Biological, Pharmaceutical, and Environmental Sciences, University of Messina, Viale F. Stagno D'Alcontres 31, I-98166 Messina, Italy

*Correspondence:

Laura De Luca, Department of Chemical, Biological, Pharmaceutical, and Environmental Sciences, University of Messina, Viale F. Stagno D'Alcontres 31, I-98166 Messina, Italy

Email: laura.deluca@unime.it

Table of contents

Figure S1. Schematic representation of the human 5-LOX protein structure.

Figure S2. Structural superimposition of human 5-LOX with human 15-LOX-2 and human 12-LOX.

S1. Structural validation of the full-length Stable-5-LOX-NDGA complex.

Figure S3. Structural validation of the full-length Stable-5-LOX-NDGA complex.

Figure S4. RMSD plot of the Stable 5-LOX–NDGA complex from the 200 ns MD simulation.

S2. Validation of the 5-LOX inhibitor pharmacophore model.

Table S1. List of compounds included in the active set for pharmacophore validation.

Table S2. List of decoys comprised in the inactive set for pharmacophore validation.

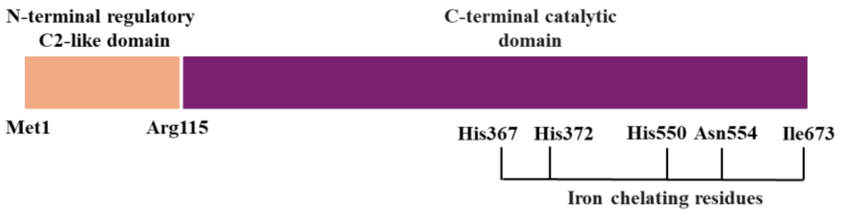

FIGURE S1. Schematic representation of the human 5-LOX protein structure. The N-terminal C2-like domain (orange) is involved in calcium and membrane binding, while the C-terminal catalytic domain (purple) contains the iron-binding site. The key iron-chelating residues His367, His372, His550, Asn554, and the terminal carboxyl group of Ile673 are located within the catalytic domain.

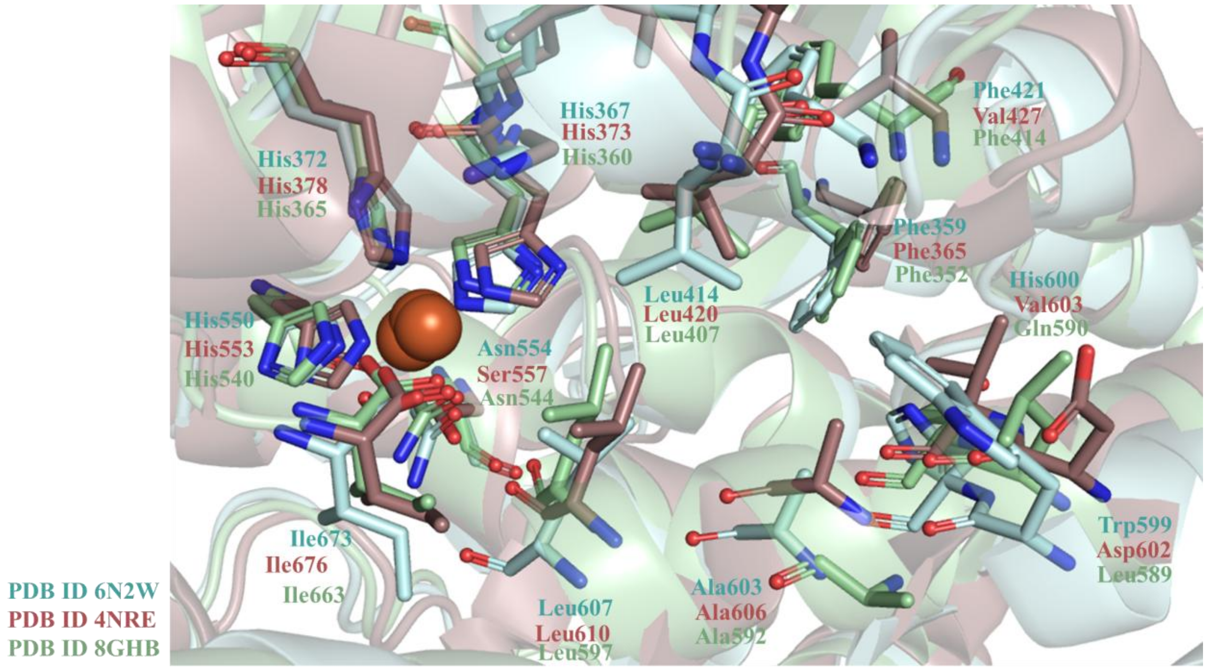

Figure S2. Structural superimposition of human 5-LOX (PDB ID: 6N2W, pale cyan) [1] with human 15-LOX-2 (PDB ID: 4NRE, dark pink)[2] and human 12-LOX (PDB ID: 8GHB, pale green) [3]. Residues lining the binding pocket are shown as sticks, while the catalytic iron ion is depicted as spheres. The remaining protein regions are represented as cartoons.

S1. Structural validation of the full-length Stable-5-LOX-NDGA complex

To validate the refined protein two tools were used: VERIFY 3D[4] and PROCHECK[5] (SAVES v6.1, Structure Validation Server (Los Angeles: UCLA-DOE LAB, 2024), https://saves.mbi.ucla.edu/). The VERIFY 3D analysis confirmed the protein structure quality, with 82.24% of residues achieving scores ≥0.1 (above the 80% threshold required for acceptance), indicating an acceptable conformation consistent with reference structures (Figure S2A). PROCHECK analysis checked the protein geometry by comparing bond angles, lengths, and dihedral angles against high-quality reference structures. The Ramachandran plot analysis revealed that 90.2% of residues occupy favorable conformational regions (exceeding the 90% threshold for good models), with 8.5% in allowed regions and no residues in disallowed regions, confirming the structural validity of the model. As illustrated by the Ramachandran plot (Figure S2B), the two triangles located in the white regions correspond to glycine residues, which exhibit greater conformational flexibility due to their lack of a side chain, allowing φ and ψ dihedral angles that would be sterically restricted for other amino acids.

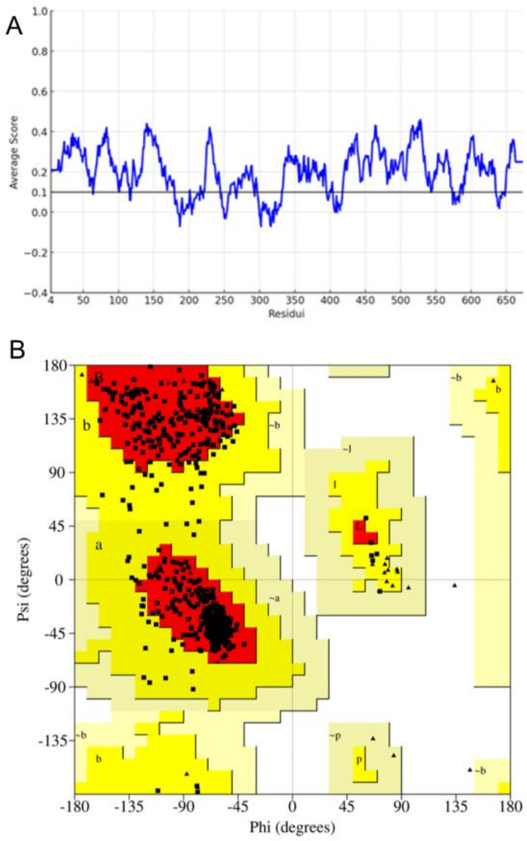

FIGURE S3. A) Graph resulted from VERIFY 3D analysis. The x-axis indicates the number of residuals and the y-axis the assigned score. We highlight the threshold set by the software of 0.1 B) Ramachandran plot obtained as an output of PROCHECK. In the diagram, the X-axis represents the φ angle, while the Y-axis represents the ψ angle. The red areas represent the favorable regions, the yellow areas the permitted regions, and the white areas the unfavorable regions.

Furthermore, to thoroughly examine the stability of the protein-ligand complex, a 200 ns molecular dynamics (MD) simulation was performed using the Desmond tool [6] from the Schrödinger Suite (Schrödinger Release 2021-4: Maestro, Schrödinger, LLC, New York, NY, 2021). The protein RMSD stabilized at 1.5–1.75 Å, indicating high structural stability, while the ligand RMSD remained consistently between 3.0–3.5 Å, demonstrating stable binding with appropriate flexibility within the active site (Figure 3S). The absence of significant fluctuations or increases in either RMSD confirms that the complex achieved a stable configuration without signs of dissociation or major conformational changes throughout the simulation period.

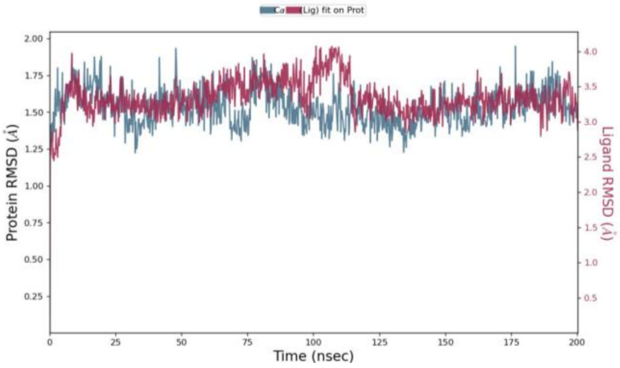

FIGURE S4. RMSD chart of the ligand-protein complex during molecular dynamic simulation. The x-axis shows the simulation time, while the y-axis shows the change in RMSD value of the ligand and protein during the trajectory

S2. Validation of the 5-LOX inhibitor pharmacophore model

The discriminatory power of the pharmacophore model was evaluated through virtual screening using a custom-designed validation set. The library comprised 11 known 5-LOX inhibitors, listed in Table S1 and retrieved from the literature [4–11], along with 500 decoy compounds generated via the LUDe web application [12] as reported in Table S2.

Table S1. List of compounds included in the active set for pharmacophore validation, along with their 2D chemical structures, reported IC_{50} values, and corresponding literature references.

Compound No.	Name	2D structure	IC50 (μM)	Reference (doi)
1	Zileuton	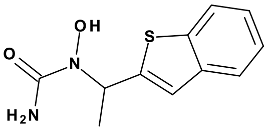	3.3	10.1016/S0960-894X(01)80717-4
2	BW70C	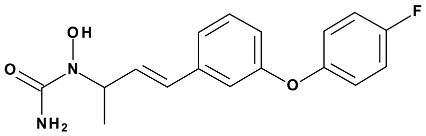	0.08	10.1016/S0960-894X(01)80717-4
3	NDGA	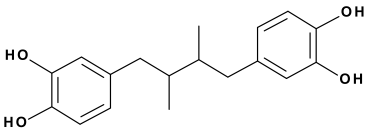	0.2	10.1016/0262-1746(84)90102-1
4	Atreleuton	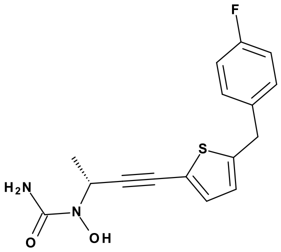	0.023	10.1021/jm9700474
5	Baicalein	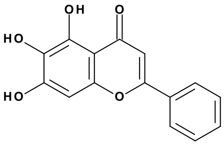	9.5	10.1007/BF01972717

6	BW A137C	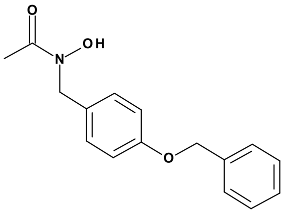	0.8	10.1021/jm00398a001
7	BW A4C	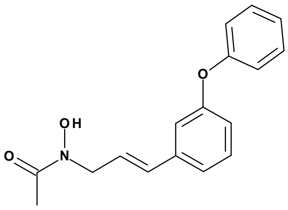	0.1	10.1021/jm00398a001
8	CAPE	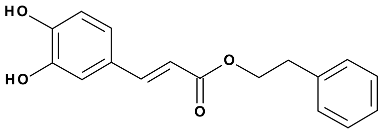	0.13	10.1371/journal.pone.0031833
9	CHEMBL119499	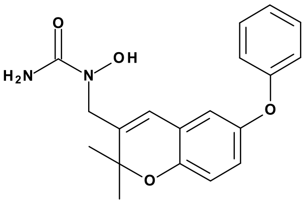	0.44	10.1021/jm00075a013
10	CHEMBL421423	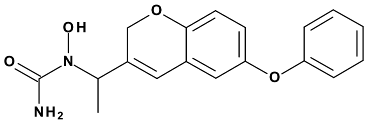	0.26	10.1021/jm00075a013
11	Honokiol	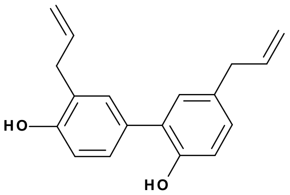	4.2	10.1016/j.bmc.2009.05.018

Table S2. List of decoys comprised in the inactive set for pharmacophore validation, along with their SMILE strings.

ID	SMILE	ID	SMILE
1	CNC(=O)c1sc(c2ccc(Cl)cc2)nc1C	251	CCN(CC)C(=O)Nc1ccc(C(=O)N(C)C)c(C)c1
2	Clc1ccc(C2(c3cc4[nH]ccnc-4n3)CC2)cc1	252	O=C1C=C(NCc2ccncc2)CC(c2ccccc2)C1
3	Cc1ccc2[nH]c(=O)n(Cc3c(F)cccc3F)c2c1	253	CCOC(=O)c1[nH]c2cc(Cl)ccc2c1C(=O)CC
4	CCc1nc(N)ncc1c1ccc(C(F)(F)F)cc1	254	c1ccc(CCc2cn(c3ccc4[nH]ncc4c3)nn2)cc1
5	O=c1[nH]c2cccnc2n1Cc1ccc2c(c1)OCO2	255	Clc1cccc(CNc2nnnn2c2ccccc2)c1
6	CN1CCC(Nc2cc(C#N)c3ccccc3n2)C1=O	256	CCCNC(=O)c1cc2c(s1)CCN2c1ncccn1
7	O=C(C1CCOCC1)N1CCCC(c2ncc[nH]2)C1	257	CCCSc1nc(O)c2cnn(c3ccccc3)c2n1
8	CC(=O)Nc1cnc(c2ccccc2C(F)(F)F)cn1	258	CC/C(=N/Nc1cccc(C)c1)c1cnnc(SC)n1
9	N#Cc1cc2c(nc1NC1CCCCC1)CCCC2=O	259	COn1cc(CC(=O)Nc2cccnc2)c2ccccc21
10	O=C(NC1CCc2ccccc21)c1cc2ccccc2[nH]1	260	Cc1ccc(CC(=O)Nc2ccc([N+](=O)[O-])cc2C)cc1
11	Cc1cc(C)n2ncc(C(=O)Nc3ccncc3)c2n1	261	CCC(C)NC(=O)c1nc(c2ccccc2F)cnc1N
12	C[C@@H]1CC[C@H]2[C@@H](C)C(=O)N[C@@H]3O[C@@]4(C)CC[C@@H]1[C@@]23O4	262	O=S(=O)(NCc1ccco1)c1ccc(Cl)s1
13	O=c1c2ccccc2[nH]c(=S)n1CC1CCCO1	263	CCCC(=O)Nc1nc2c(OC)ccc(OC)c2s1
14	Nc1ncnc2c1C1(CCCCC1)Cc1ccccc1-2	264	COCCCNc1ccn2nc(c3ccccc3)cc2n1
15	C=C1C(=O)OC2C1CC(O)/C(C)=C\CCC1(C)OC21	265	COc1ccc(C(=O)Nc2cccc(Cl)c2)cc1OC
16	O=C(NN1CCOCC1)c1ccc(Cl)c(Cl)c1	266	COc1ccccc1NC(=O)/C(C#N)=C/c1ccncc1
17	Cc1cccc2c(O)nc(c3ccc([N+](=O)[O-])cc3)nc12	267	COc1cccc2c(N/N=C/c3ccco3)cc(C)nc12
18	Nc1ncc2c(n1)CCN(c1ccc([N+](=O)[O-])cc1)C2	268	CCc1cccc(C)c1N(C(=O)CCl)C(C)CCO
19	Cn1cc(c2cnc3ncc(c4cn[nH]c4)cn32)cn1	269	O=C(CCNc1cccc(F)c1)c1cccc([N+](=O)[O-])c1
20	Cc1c(NC(=O)c2noc3c2CCCC3)cnn1C	270	Cc1ccc(NC(=O)CCn2nnc3ccccc32)cc1
21	N#CCC(=O)NN=C1CCCCCCCCCCC1	271	OCCNc1nc(Nc2ccccc2)nc2ccccc12
22	Cc1cccn2c(/C=N/O)c(c3ccc(F)cc3)nc12	272	COc1ccc(c2csc(Cc3[nH]cnc3C)n2)cc1
23	C[S+]([O-])c1cc(c2cccs2)[nH]c(=O)c1C#N	273	Cc1ccc(CNS(=O)(=O)c2ccc(C)s2)cc1
24	CNC(=O)C(C)c1ncc(C(F)(F)F)cc1Cl	274	COc1ccc(S(=O)(=O)Nc2cc(C)ccn2)cc1
25	CC(C)(C)c1nnc(NC(=O)c2cccs2)s1	275	Cc1cc(NCc2ccccc2)nc(N2CCOCC2)n1
26	CC1C(=O)O[C@@H]2[C@H]1CC[C@H](C)[C@@]1(O)CCC(=O)[C@@]21C	276	Cc1nc(C(C)C)sc1C(=O)NCCn1ccnc1
27	COc1nc(NC(C)=O)nc(C)c1Br	277	O=C(COc1ccc(Br)cc1F)NC1CC1
28	Cc1cc2c(nc1C(O)c1cccnc1)CCCCC2	278	Cc1ccc(CNc2ccc(N3CCOCC3)cc2)cc1
29	c1ccc(C2CCCN2c2ccnc3[nH]ncc23)cc1	279	Oc1cccc(CCNc2ccnc3ccccc23)c1O
30	Cc1nc(O)c2c(Sc3ccccn3)cccc2n1	280	Cc1ccccc1NC(=O)/C=C/c1ccc([N+](=O)[O-])s1
31	CC(C)(C)/[N+]([O-])=C/c1c[nH]c(c2ccc(Cl)cc2)n1	281	C[C@@H]1C[C@H]1c1nc(NC2CC2)cc(N2CCCCCC2)n1
32	Cc1cc(NC(=O)C23CCC(C)(C(=O)O2)C3(C)C)no1	282	O=C(NCCO)Nc1ccc(Cl)cc1n1cccc1
33	O=c1[nH]c2c(nc3ccc(Cl)cn32)c2ccccc12	283	Cc1ccc(CNc2ccccc2n2ccc(C)n2)nc1
34	O=C(Nc1cc(Cl)cc(Cl)c1)c1ccccn1	284	COC(=O)c1ccc(/C=N/Nc2nc(C)c(C)s2)cc1
35	O=C1CCC(Cc2ccc(Cl)cc2Cl)C(=O)N1	285	COc1cc2ncc(NC3CCCCC3)nc2cc1OC
36	S=C(N/N=C1\CCc2ccccc21)Nc1ccccc1	286	Cc1cc(NCCO)nc(Nc2ccc(F)c(F)c2)n1
37	Cc1cc2nc(/C(C#N)=C/c3ccncc3)[nH]c2cc1C	287	CCC1Sc2ccccc2N(CC(=O)NC2CC2)C1=O
38	Cc1[nH]ncc1c1cn2c(cn1)ncc2c1cn[nH]c1	288	Cc1ccc(Cn2c(CCCO)nc3ccccc32)cc1
39	CC1(C)C(c2ccccc2)=NC(c2ccccc2)N1O	289	Cc1cc(C)cc(C(=O)OCC(=O)NCc2ccco2)c1
40	COc1cc2c(cc1c1nc3c(cnn3C)[nH]1)OCO2	290	COc1ccc(OC)c(NC(=O)/C=C/c2ccc(C)o2)c1
41	Cc1n[nH]c(C(F)(F)F)c1N=NN1CCOCC1	291	Cc1ccc(NC(=O)/C=C/c2ccc([N+](=O)[O-])s2)cc1
42	C=CCn1c(=S)[nH]c2sc3c(c2c1=O)CCC3	292	CC(C)(Oc1ccc(Cl)cc1)C(=O)Nc1ccncc1
43	Cn1nnc(NC(=O)c2ccc(C(C)(C)C)cc2)n1	293	Cc1ccc(S(=O)(=O)Oc2ccccc2/C=N/O)cc1
44	COc1cc(Cl)ccc1c1nc2cc[nH]cc-2n1	294	Cc1ccc(S(=O)(=O)Nc2ccc(N(C)C)nc2)cc1
45	C[C@H]1C=C2N3CCC[C@@H]4C(=O)C[C@H](C1)C24[C@H](O)CC3	295	Cc1ccc(S(=O)(=O)NCc2csc(C)n2)cc1

46	C[C@H]1CC[C@@]2(C)[C@@H](CC=C(C=O)[C@]2(O)C=O)C1(C)C	296	COC(=O)c1ccc(NC(=O)c2ccc(OC)cc2)cc1
47	O=C(Nc1ccc(Cl)cc1)c1cnn2ccccc12	297	CC(Nc1ccc(F)cc1)c1nnnn1c1ccccc1
48	O=C1NCCc2nc(C#Cc3ccccc3F)sc21	298	COc1ccc(c2cc(c3ccc(CO)cc3)on2)cc1
49	CC(=O)Nc1ccc2c(c1)ncn2c1ccc(C)cc1	299	O=C(CCc1cscn1)N1CCCC(c2ccn[nH]2)C1
50	O=C1Nc2ccccc2C(=O)N2Cc3ccccc3C[C@@H]12	300	Clc1ccccc1CCSc1ncnc2[nH]cnc12
51	CC[C@@]12CCCN3C(=O)C[C@@]4(O)c5ccccc5N(C(=O)CC1)C324	301	O=C1c2ccccc2C(=O)c2sc(Nc3cccc(Cl)c3)nc21
52	Cc1ccsc1C1C2=C(O)CC(C)(C)CC2=Nc2ncnn21	302	CC[C@H](C)[C@H](NC(=O)/C=C/c1ccc(Cl)cc1Cl)C(=O)OC
53	CC(C)c1ccc(NC(=S)N(C)CCc2ccccn2)cc1	303	CCOc1ccc2c(c1)c(CCNC(=O)CC)c1n2Cc2ccccc2-1
54	CS(=O)(=O)CCSc1n[nH]c(c2ccc3c(c2)CCC3)n1	304	O=C1NC2(CCCCC2)C(=O)N1/N=C/c1ccccc1Br
55	COc1ccccc1NC(=O)CCn1c(=S)oc2ccccc21	305	COc1ccc(C(=O)Nc2ccc(Cl)cc2)cc1OC1CCCC1
56	c1ccc(CCn2nnc3c(NC4CCCC4)ncnc32)cc1	306	O=C(NC(=S)N1CCN(c2ccc(F)cc2)CC1)c1cccs1
57	Cc1nn(c2ccccc2)c2c1c1c(c3cccn32)C(=O)NC1=O	307	CC1(C)CC(C(=O)NCCC(c2ccccc2)c2ccco2)CCO1
58	COc1cccc(CSc2nnnn2c2ccc(O)cc2)c1	308	Cc1ccc(Cl)cc1N1CCC(CNC(=O)CCn2ccnc2)C1
59	C/C(=N\NC(=O)[C@@H]1C[C@H]1c1ccccc1)c1ccc([N+](=O)[O-])cc1	309	COC(=O)N1CCC[C@@]2(CCN(C(=O)Nc3ccc(OC)cc3)C2)C1
60	O=C(CSc1ccccn1)Nc1ccc(Cl)c([N+](=O)[O-])c1	310	O=C1NC(=O)/C(=C\c2ccc(c3ccc(Cl)c(Cl)c3)o2)S1
61	O=C(c1c(O)cc(Cl)cc1Cl)N1CCCC2(CCC2)C1	311	O=C1NC(=O)C(CC2COc3c(Cl)cc(Cl)cc3C2=O)S1
62	O=S(=O)(NCCc1ccccc1)c1cccc2nsnc12	312	CCC(CC)Nc1nc(C)nc(C(=O)c2c(C)cc(C)cc2C)c1C
63	O=C(Cn1cncn1)Nc1ccc(Oc2ccc(Cl)cc2)cc1	313	c1ccc(CCCNc2ncnc3oc(c4ccccc4)nc23)cc1
64	CCOC(=O)c1cn(CC(=O)Nc2ccccc2)c2ncccc12	314	Cc1nc2c(c3ccccc3)c(c3ccc(O)cc3)nn2c(C)c1C
65	CCOC(=O)N1CCC(NS(=O)(=O)c2ccc(C)cc2)CC1	315	Cc1cccc(c2cc(NCCCn3ccnc3)c3ccccc3n2)c1
66	O=C(CC(=O)c1ccccc1)Nc1cc([N+](=O)[O-])ccc1Cl	316	Cc1nc(N2CCCCC2)c2[nH]c(C34C[C@H]5C[C@@H](C3)C[C@@H](C4)C5)cc2n1
67	CCOC(=O)C1=C(C)NC(C)=C(C(=O)OCC)C1c1ccco1	317	CC(Cc1cccs1)C(=O)NCc1cccc(N2CCCC2=O)c1
68	CC(=O)NCC1CN(c2ccc(C(C)=O)c(Cl)c2)C(=O)O1	318	O=C(c1cccc(Cl)c1)N1CCC(c2nc3ccccc3[nH]2)CC1
69	Cc1ccc(c2noc(CCC(=O)Nc3ccccc3C)n2)cc1	319	COc1cc(/C=C/C=C/C=C/C=C/c2[nH]ccc2Cl)oc(=O)c1C
70	O=C(CSc1nnc(c2ccco2)o1)NC1CCCCCC1	320	O=C(COC(=O)CC1CC2CCC1C2)NCc1ccc(Cl)cc1
71	COc1ccc(CCc2c[nH]c(=O)c3cncnc23)cc1OC	321	CCC(=O)NCC(=O)N(C)C(c1ccc(Cl)cc1)c1cccnc1
72	COc1c(Cl)c(O)nc(C(Cl)(Cl)Cl)c1Cl	322	O=C(Nc1ccc2nc3ccccc3nc2c1)c1ccccc1Cl
73	CCC1CCCCN1C(=O)c1ccc(C)nc1NCc1ccco1	323	O=C1c2ccc(Nc3ccc(F)cc3F)cc2OCc2ncccc21
74	COC[C@H](C)Nc1ncnc2c1cnn2c1ccc(Cl)cc1	324	Cc1onc(c2cccnc2)c1COc1ccc(C(=O)NC2CC2)cn1
75	COc1cccc([C@H](C)Nc2cc(c3ccncc3)nc(C)n2)c1	325	CC(=O)N[C@@H](C)c1ccc(C2CN(c3ccc(OC4CC4)cc3)C2)cc1
76	O=C(CSc1nc[nH]n1)N/N=C/c1ccc(C(F)(F)F)cc1	326	O=C(NC12CCCC(C#Cc3cccc(F)c3)(CC1)C2)c1cnccn1
77	O=[N+]([O-])c1cnc(/C=C/c2cccc3ccccc23)n1CCO	327	CN(C)c1ccc(C(C/C(=N\O)c2ccncc2)c2ccccc2)cc1
78	CNC(=O)CSc1n[nH]c(c2cc(Cl)ccc2OC)n1	328	Cc1ccc(C)c(COc2cc([N+](=O)[O-])ccc2NS(C)(=O)=O)c1
79	CCC1(C(=O)NCCc2ccc(C)cc2)C(C)C1(Cl)Cl	329	O=C1NC(=O)/C(=C/c2ccc(c3cccc(Br)c3)o2)S1
80	CCS(=O)(=O)c1ccc(CC(=O)Nc2nccs2)cc1	330	COc1ccc(C2c3ccsc3CCN2C(=O)NC(C)(C)C)cc1
81	Cn1c(SCC(=O)Nc2ccc(Cl)cn2)nnc1C1CC1	331	CC(=O)C[C@@]1(O)C(=O)C2=C(OC[C@@H]2C)c2ccc3c(C)cccc3c21
82	O=C(c1ccc2nccn2c1)N(CCO)Cc1ccccc1F	332	Cc1nccc(C(=O)N[C@]23CCC[C@](C#Cc4ccccn4)(CC2)C3)n1
83	CCOc1ncccc1CNC(=O)N1CCCC1c1ccncc1	333	Cc1cc(O)c(c2ccnc3cc(c4ccccc4)nn32)cc1Cl
84	CCCS(=O)(=O)NC(=O)c1ccc(n2nc(C)cc2C)cc1	334	COCCn1cc(C(=O)Nc2ccc3ccccc3c2)c2ccccc21
85	CNc1nc(SCc2ccccc2)nc(N2CCOCC2)n1	335	Cn1cc(c2ccc(c3nc4cc(C(F)(F)F)ccc4[nH]3)cc2)cn1
86	Cn1c(SCC(=O)N/N=C/c2ccncc2)nc2ccccc21	336	CCOCn1c(Sc2cc(C)cc(C)c2)c(CC)c(=O)[nH]c1=O
87	COc1cc(C(=O)Nc2ncc(C)s2)cc(OC)c1OC	337	Cc1cnc(c2cnn3c(=O)cc(c4ccc5c(C)noc5c4)[nH]c23)o1
88	COC(=O)c1cc(OC)c(OC)cc1NC(=O)c1cccc(C)c1	338	Cc1cc(c2ccccc2)ncc1C(=O)Nc1ccc2cccnc2c1
89	COc1ccc(c2cc(c3cc(OC)ccc3OC)n[nH]2)cc1	339	COC(=O)c1ccc2c(=O)n(c3ccccc3F)c(=S)[nH]c2c1
90	N#C/C(=N\Nc1cccc(Cl)c1)C(=O)c1cc(C2CC2)on1	340	Cc1ccc(n2nc3c(c2NC(=O)C(C)(C)C)CSC3)c(C)c1
91	Cc1cc([C@H]2Oc3cc(O)cc(O)c3C[C@@H]2O)c(C)c(O)c1O	341	O=C(CCC1CCCCC1)Nc1ncc(Cc2ccccn2)s1

**92**	CC1COc2ccccc2N1C(=O)N1CCC(c2c[nH]cn2)CC1	**342**	CC[C@H]1Cn2nc(c3ccc(Cl)cc3Cl)c3nc(C)cc(c32)N1
**93**	C=CC1=C(C(=O)NCCC)C(c2ccc(Cl)c(Cl)c2)OC1	**343**	COc1cccc(c2cc(O)c3c(Br)ccnc3n2)c1
**94**	Cc1nc(NC(=O)CSc2nnc(C3CC3)n2C)sc1C	**344**	CCOC(c1ccc(N(C)C)cc1)c1ccc(C(C)C)cc(=O)c1O
**95**	COc1cccc(CSc2nnc(c3ccc(C)cc3O)o2)c1	**345**	Cc1c2c(ccc1c1cc(=O)n3ncc(c4ccccn4)c3[nH]1)OCO2
**96**	Cc1ccc(n2nnnc2C/C=N/Nc2ccc(Cl)cc2)cc1	**346**	COc1ccc(/N=C2\C(=O)Nc3ccc(Br)cc32)cc1
**97**	CN(C)S(=O)(=O)c1cc(NC(=O)CCCl)ccc1Cl	**347**	O=C(Nc1ccccc1C(F)(F)F)Oc1ccc2cccnc2c1
**98**	COc1ccc(Nc2nnc(c3nsc4ccccc43)o2)cc1	**348**	CC(=O)NC[C@H]1CC[C@]2(CC1)OO[C@]1(O2)C2CC3CC(C2)CC1C3
**99**	CC(C)NC(=O)OCC[S+]([O-])c1c(Cl)c(Cl)nn1C	**349**	O=C1NC(=O)/C(=C\c2ccc(c3cccc(Br)c3)o2)S1
**100**	COC(=O)C(NC(=O)c1ccc2oc(C)nc2c1)C(C)C(C)C	**350**	O=C(NCCCCn1ccnc1)c1sc2ccccc2c1Cl
**101**	COc1ccccc1/C(C#N)=C/c1ccc(N(C)C)cc1	**351**	CCCCN1C(=O)/C(=C/NC(C)(C)CC)C(=O)c2cccc(C)c21
**102**	C1=CC2C(c3ccnc(Nc4ccccc4)n3)=CNN2C=C1	**352**	Cc1ccc(C(=O)NC(=S)Nc2ccc([N+](=O)[O-])cc2O)cc1
**103**	C/C(=N/NC(=O)c1ccccn1)c1c[nH]c2ccccc12	**353**	O=[N+]([O-])c1ccc2nc(c3ccc(Br)cc3)[nH]c2c1
**104**	COc1cccc(c2nc(c3ccccn3)no2)c1OC	**354**	COc1ccc(Cn2ccc3c4c(N)nc(N)nc4ccc32)cc1
**105**	Cc1cccc(c2noc(CSc3ccccc3)n2)c1	**355**	O=C(Nc1ccccc1C(F)(F)F)c1ccc2nccnc2c1
**106**	CCCc1cc(O)nc(Nc2ccc3c(c2)OCCO3)n1	**356**	CNc1ccnc2sc3c(=O)n(c4ccc(C)cc4)ccc3c12
**107**	CCCc1cc(c2nnc(S)n2CC(C)C)cs1	**357**	COc1ccc(COC(=O)CNC(=O)C2CCCCC2)cc1F
**108**	Cc1ccc(NC(=O)CCc2c[nH]c3ccccc23)nc1	**358**	CC#CC(=O)Nc1ccc(Cl)c(c2nc3cc(C)ccc3o2)c1
**109**	CCOc1ccc(C(=S)N2CCCC2)cc1OCC	**359**	CCCc1cc2c(cc1Cl)N(C(=O)Nc1cccnc1)CC2
**110**	CCCOCCCNC(=S)Nc1cc(F)ccc1F	**360**	O=c1nc(SCc2ccccc2)nc(SCC2CCC2)[nH]1
**111**	Cc1ccc(C(=S)NCc2ccc(Cl)cc2)c(O)c1	**361**	Cc1ccc2[nH]c(n3[nH]c(C)c(Cc4ccccc4)c3=O)nc2c1C
**112**	Cc1ncsc1C(=O)N(Cc1cccs1)C1CC1	**362**	Cc1ccccc1n1ccnc1SCC(=O)NCC1CCCO1
**113**	c1nc(CCCn2cc(CC3CCCCCC3)nn2)c[nH]1	**363**	Cc1nc2ccccn2c1C(=O)Nc1cccc(C(F)(F)F)c1
**114**	COc1ccc(C(S)=Nc2ccc(N(C)C)cc2)cc1	**364**	O=C1NS(=O)(=O)N(Cc2ccccc2Cl)c2ccccc21
**115**	Cc1ccc(C(=O)NCCc2c[nH]c3ccccc23)s1	**365**	Nc1nccn2c(C3CCC3)nc(c3ccc4sccc4c3)c12
**116**	Clc1ccccc1CO/N=C1\CCCc2nonc21	**366**	Cc1c(OCC2CC2)ccnc1CSc1nc2ccccc2[nH]1
**117**	C=CC(C)(C)c1c(OC)oc2ccc(OC)c(C)c2c1=O	**367**	CC1(C)Oc2ccc(C#N)cc2[C@@H](Oc2ccc(S)nn2)[C@@H]1O
**118**	CCCCOP1(=O)CC(C)=C(Cl)C(C)(OC)C1	**368**	NC(=O)c1cnc2[nH]ccc2c1NC1C2CC3CC1CC(F)(C3)C2
**119**	Cc1ccc(C2=CC=C(C=O)C(C)(c3ccc(C)o3)C2)o1	**369**	Cc1nc(N2CCCCC2)c2[nH]c(c3cc(F)ccc3F)cc2n1
**120**	CCOC(=O)c1c(N)scc1c1cccc([N+](=O)[O-])c1	**370**	COc1cc(/C=C/c2nc(O)c3ccccc3n2)cc(Cl)c1O
**121**	CCCCc1cc(OC)c2sccc2c1OC(C)=O	**371**	Cc1ccccc1C(=O)Nc1cccc(NC(=O)c2cccnc2)c1
**122**	Oc1ccc(Cc2nnc3ccc(c4ccc[nH]4)cn23)cc1	**372**	COc1ccc(c2nc(SC)[nH]c2c2ccc(OC)cc2)cc1
**123**	C=C[C@H](OC(=O)NCC)c1ccc(OC(=O)NCC)cc1	**373**	O=c1[nH]c(CSC(=S)NCc2ccco2)nc2ccccc12
**124**	COC(=O)/C(=C/c1ccc(Cl)cc1Cl)CN=[N+]=[N-]	**374**	C/C=C/COC1(C(F)(F)F)OC(=O)Nc2ccc(Cl)cc21
**125**	COCOc1ccccc1C1CC(=O)c2ccccc2O1	**375**	O=C(c1cc(c2ccc(Cl)cc2Cl)[nH]n1)N1CCCCC1
**126**	CCCc1cc(c2nc(c3cncnc3)cs2)ccn1	**376**	Nc1nc(N)c2cc([S+]([O-])Cc3ccc(Cl)cc3)ccc2n1
**127**	C=CC(=O)Nc1ccc(Cl)cc1C(O)c1ccccc1	**377**	Cn1c(=O)[nH]c2cc(c3noc(c4ccc(C#N)cc4)n3)ccc21
**128**	C=C(c1cc(OC)c(OC)c(OC)c1)c1cccs1	**378**	O=C1C/C(=C/c2ccc(c3ccc4c(c3)COC4=O)s2)C(=O)N1
**129**	COc1ccc(c2sncc2c2ccc(C)cc2)cc1	**379**	CCCOc1cc(C(=O)Nc2c(F)cccc2F)ccc1OC
**130**	Cc1ccc(CN(C)c2ccc([N+](=O)[O-])cc2Cl)o1	**380**	CC(C)=CC(=O)OCC(=O)Nc1ccc(SC(F)F)cc1
**131**	Cc1cccc(OCc2nc(C#N)c(N3CCCC3)o2)c1	**381**	O=C1/C(=C/c2nc3ccccc3[nH]2)SC(=S)N1C1CCCC1
**132**	C=C(C)c1cccc(C(C)(C)NC(=O)NC2CCCC2)c1	**382**	COC(=O)[C@@]1(C)CCC[C@]2(C)c3cc(OC)c(O)cc3CC[C@@H]12
**133**	CN(C)/C=N/c1cc(c2ccccc2)nn1c1ccccc1	**383**	Cc1cnc(NC(=O)c2ccc(NC(=O)CC(C)(C)C)cc2)s1
**134**	C=CCNc1nc(c2c[nH]c3c(CC)cccc32)cs1	**384**	CNC(=O)c1cc(c2ccc(Cl)cc2Cl)c(C#N)c(C)n1
**135**	COc1ccc(C(=O)c2ccc(C)cc2)c(OC)c1OC	**385**	CC1Cc2nc(S)nc(O)c2C(c2ccc(C(C)C)cc2)O1
**136**	CCCCCOc1ccc2c(c1)CCc1sc(N)nc1-2	**386**	COc1ccc2c(n1)[nH]c(c1ccc(F)cc1)c2c1ccncc1
**137**	c1csc(CNc2nccc3[nH]c4ccccc4c23)c1	**387**	CC1CCC(C(C)C)C(OC(=O)CSc2nnc(N)s2)C1
**138**	COCc1cc(Sc2ccc(Cl)cc2)nc(C)n1	**388**	CCNC(=O)NC1CCCc2c1cncc2c1ccc(C#N)cc1

**139**	CCCCCCOc1nc(c2ccncc2)cc(=O)n1C	**389**	Cc1cccc(/C=N/NC(=O)c2cnc3c(F)cccc3c2O)c1
**140**	COCCOc1cc(n2cccc2)c(Cl)cc1Cl	**390**	O=C1Nc2ccc(Cl)cc2[C@@]12C[C@@H]2c1cccc(c2ccco2)n1
**141**	CCc1ccc(NC(=O)c2[nH]c(C)c(C(C)=O)c2C)cc1	**391**	Cc1ccc(NC(=O)c2cnc(Cl)nc2C(F)(F)F)s1
**142**	CSCCc1nc(C(C)C)nn1c1ccncc1C	**392**	COc1cc(F)ccc1c1cc(C(=O)NCC2CCCCC2)[nH]n1
**143**	O=C(Nc1ccccc1)Nc1cc([N+](=O)[O-])ccc1F	**393**	COc1cc(CNc2nc(C(F)(F)F)no2)cc(OC)c1OC
**144**	CN(Cn1c(=O)oc2ccc(Cl)cc21)c1ccccn1	**394**	OCc1cccc(Nc2nccc(Nc3cccc4[nH]ncc34)n2)c1
**145**	COC(=O)CC(c1ccc(C#N)cc1)n1c(C)ccc1C	**395**	CCc1ccc(S(=O)(=O)NCCc2cn3ccccc3n2)s1
**146**	COc1ccc(Cl)cc1NC(=O)NCc1ccco1	**396**	O=C(NNC(=O)c1ccc(n2cccc2)cc1)c1ccc(F)cc1
**147**	COc1ccc(c2nc(C3CCCCC3)no2)cc1OC	**397**	CC1(S(=O)(=O)c2cccc3ccccc23)SC(=O)NC1=O
**148**	COc1ccc(Cc2nc(c3cccc(C)c3)no2)cc1	**398**	O=C(NO)C(c1ccc(c2ncccn2)cc1)c1ccccc1F
**149**	Cc1ccc(SCc2nc(c3ccccn3)no2)cc1	**399**	O=C1C=C(N2CCC(c3ccccc3)CC2)C(=O)c2c(O)cccc21
**150**	CSCc1cc(F)ccc1CNC(=O)N1CCCC1	**400**	CCNC(=S)N1CCN(c2ccnc3cc(Cl)ccc23)CC1
**151**	O=C(CSc1c[nH]c2ccccc12)NC1CCCC1	**401**	c1ccc2c(Nc3nc(C4CC4)cs3)ncnc2c1
**152**	CCOc1c2ccoc2nc2c(OC)c(OC)ccc12	**402**	C[C@H]1CCC(=O)N([C@H](CC#N)Cc2ccccc2)CC1
**153**	O=C(CNC(=O)c1ccco1)Nc1cccc(Cl)c1	**403**	COc1cccc(c2nc(c3cccnc3)ns2)c1
**154**	COc1nn([C@H](C)c2ccc(CC(C)C)cc2)c(=O)o1	**404**	Cc1cccc2nc(N/N=C/c3ccco3)sc12
**155**	CC(C)CCN(C)c1ccc(/C=N/n2cnnc2)cc1	**405**	CCC1=C[C@@](CC)(C[C@H](/C=C/C(C)=O)CC)OC1=O
**156**	CCn1cc(CNC(=O)NC2CCCCC2)c(C)n1	**406**	CCCCCCNc1nc(SC)nc2ncccc12
**157**	CCCCC(=O)c1ccc2c(c1)sc(=O)n2C(C)=O	**407**	C/C(=N\Nc1nccnc1Cl)c1ccc(F)cc1
**158**	CCNc1nc2cc(Cl)c(C#N)cc2nc1NCC	**408**	CC(C)(C)c1csc(N/N=C/c2cccnc2)n1
**159**	CCOP(=O)(/N=C1\SCC(C)S1)OCC	**409**	CNc1oc(Cc2cccc3ccccc23)nc1C#N
**160**	CCOn1c(SC(C)C)nc2ccccc2c1=O	**410**	O=C(Cc1ccncc1)OC12CC3CC(CC(C3)C1)C2
**161**	CCCCCC1(CC(C)(C)C)C(=O)NC(=O)NC1=O	**411**	COC(=O)/C(=C/c1ccccc1Cl)CSC#N
**162**	CCC(CC)C(=O)Nc1nc2c(s1)CC(C)CC2	**412**	CC(C)CC1SCC(=O)Nc2c1cnn2C(C)C
**163**	C=CCn1c(SCCC#N)nnc1c1ccncc1	**413**	COc1cccc(Oc2ccnc3ccsc23)c1
**164**	Cc1ccc(c2ccc(COC3CN(C#N)C3)cc2)cc1	**414**	O=C1c2ccccc2C(=O)N1CCC1=CCCCC1
**165**	OCCNc1nc2ccccc2c(c2ccccc2)n1	**415**	CCCCCCCCn1cnc2ccc(C)cc2c1=O
**166**	COc1ccc(/C=C(\C#N)c2ccccn2)cc1OC	**416**	COc1ccc(Cl)cc1NC(=O)c1ccoc1C
**167**	O=C(NCCCl)Nc1ccc(C2CCCC2)cc1	**417**	COCn1c2ccccc2nc1c1ccc(OC)cc1
**168**	Cc1cccc(CCNc2cc(C)nc3ncnn23)c1	**418**	c1coc(Cn2c3ccccc3nc2c2ccco2)c1
**169**	CCCCCCN1C(=O)COc2cc(Cl)ccc21	**419**	COc1cnc(c2ccccc2)c(c2ccccc2)n1
**170**	C=C(C(=O)OC)C(OC(C)=O)c1ccc(Cl)cc1	**420**	CNC(=O)c1ccc(Nc2c[nH]c3ccccc23)cc1
**171**	CC(Cc1cccs1)NC(=O)NC1CCCCC1	**421**	O=C(CC1OC(=O)c2ccccc21)c1cccc(F)c1
**172**	COCCOC1CCN(c2nccc3occc23)CC1	**422**	C=C(c1ccc(OC)cc1)C1COC2(CCCC2)OO1
**173**	c1ccc(CSc2nncn2c2ccccc2)cc1	**423**	O=[N+]([O-])c1cc(F)cc(F)c1Nc1ccc(F)cc1
**174**	C[C@@H](NC1=C(Nc2ccncc2)C(=O)C1=O)C(C)(C)C	**424**	COc1cccc(/C=C/c2nc(C)c(C)nc2C)c1O
**175**	O=[N+]([O-])c1ccc(NCCCO)c(C(F)(F)F)c1	**425**	Cc1ccc(C(=O)/C=C/c2ccccc2Cl)cc1
**176**	COc1cc(OC)cc(/C(C#N)=C/c2ccccn2)c1	**426**	O=C(OCC1CCCCC1)c1[nH]nc2ccccc12
**177**	CCCOC(=O)c1c(N)sc(C(=O)OCC)c1C	**427**	CN(C)c1ccc(/C=C/c2ccc3nonc3c2)cc1
**178**	CCCCCCC1C(C#N)=C(N)OC2=C1C(=O)CCC2	**428**	Cc1cc(N/N=C/c2ccncc2)nc2ccccc12
**179**	COc1ccc(c2cccc(OC)c2OC)cc1OC	**429**	CSc1nnc(C)c(/C=C/Nc2ccc(F)cc2)n1
**180**	CCOC(=O)c1cc(c2cccs2)nn1CCC#N	**430**	Sc1nnc(C2CCCCC2)n1Cc1ccccc1
**181**	CCOCc1cn(c2cc(Cl)cc(Cl)c2)nn1	**431**	Clc1ccc(OCCn2ccnc2)cc1Cl
**182**	CCCCCCC(C)n1[nH]c(=O)c2c(C)cc(=O)[nH]c21	**432**	CCOc1ccc(N(C)c2ncnc3c2CCC3)cc1
**183**	N#Cc1cccc(CSC(C(N)=O)C2CCCC2)c1	**433**	Cc1ccnc(Nc2cc(C)nc(c3ccccc3)n2)c1
**184**	COc1ccc(c2nnc(NC(=O)C3CC3)s2)cc1	**434**	Cc1cc2c(N/N=C\c3cccs3)ncnc2s1
**185**	O=C(CCc1nc2ncccc2[nH]1)Nc1ccccc1	**435**	COc1cc(/N=C/c2ccccc2O)cc(OC)c1

186	COc1ccc(c2nc(CCl)cs2)cc1OC	436	COc1ccccc1C=NN=Cc1ccccc1OC
187	COc1ccc(CSc2nnc(N)s2)cc1F	437	N#Cc1ccc(Cn2cncc2c2ccccc2F)cc1
188	C#CCCCC(=O)N1C(=O)CC1Sc1ccccc1	438	COc1cccc(c2n[nH]c(c3ccc(C)cc3)n2)c1
189	O=C(N/N=C/Nc1cccc(Cl)c1)c1ccncc1	439	Cc1ccc(CSc2ncnc3onc(C)c23)cc1
190	CC(C)CCSc1cccc(OS(C)(=O)=O)n1	440	COc1ccc(c2csc(c3ncccn3)n2)cc1
191	CCOc1cccc(/C=N/NC(=O)C2CCCC2)c1O	441	CN(N=O)c1ccc(C=C2C=Cc3ccccc32)cc1
192	C=C(Cn1ccccc1=O)C(=O)c1ccc(Cl)cc1	442	COc1cc(/C=C\c2ccncc2)cc(OC)c1OC
193	COC(=O)[C@H]1[C@H](/C=C\c2ccc([N+](=O)[O-<br>])cc2)C1(C)C	443	COc1ccnc(CSc2nc3ccccc3[nH]2)c1
194	CCOC(=O)C[C@H]1CCC(=O)/C1=C/C1CCCCC1	444	C#CCn1ccc2ccc(OC(=O)N(CC)CC)cc21
195	C/C=C/C1=CC(=O)C[C@H](c2ccc(OC)c(OC)c2)C1	445	COc1ccc(Sc2ccccc2[N+](=O)[O-])cc1
196	Cc1cc(C)nc(SCc2ccc([N+](=O)[O-])cc2)n1	446	CCCc1cc(C(=O)Nc2nnc(C)s2)cs1
197	COc1ccccc1OCCCn1c(C)c(C)oc1=O	447	Cc1ccc(Cn2c(O)c(N=O)c3ccccc32)cc1
198	Cc1nc2cccc(CCc3ccccc3)n2c1CC#N	448	O=C(CCCCCCc1cccnc1)N1CCCC1
199	C=CCn1c(S)nnc1c1cccc(OC)c1OC	449	COc1ccc(Cc2nc3cc(Cl)ccc3o2)cc1
200	[O-][S+](CCC(F)=C(F)F)c1nc2ccccc2o1	450	Cc1ccc(c2cncn2Cc2ccc(C#N)cc2)cc1
201	Clc1cccc(n2cc(c3cccc(C4=NN=NN4)c3)nn2)c1	451	C=CCn1nc(C)c(c2ncnc3[nH]ccc32)n1
202	CC(NC(=O)C(C#N)C(C)(C)C)c1ccc(Cl)c(Cl)c1	452	COc1cnc(c2ccn[nH]2)nc1NC(C)C
203	CC(NC(=O)c1ccncc1)c1ccc(Br)cc1	453	N#Cc1cccc(NC(=O)CCn2ccnc2)c1
204	O=C(N/N=C/c1ccc([N+](=O)[O-])o1)C1C2CC3CC(C2)CC1C3	454	O=C(N/N=C/C=C\c1ccco1)c1ccccn1
205	O=[N+]([O-])c1c(Nc2cc(F)cc(Cl)c2)ccc2nonc12	455	C/C(=C/CC[C@@]1(C)[C@@H]2CC[C@@H](C2)[C@@]1(C)O)CO
206	CC12CCC(C(=O)NCc3ccccc3Cl)(OC1=O)C2(C)C	456	CC(C)Cn1cnc2c(NC3CC3)ncnc21
207	O=C1Nc2ccccc2C(c2ccccc2)=N[C@H]1Cc1ccccc1	457	CC(C)=CCNc1ncnc2c1ncn2C(C)C
208	c1ccc(c2nc(c3ccncc3)sc2C2=NNN=N2)cc1	458	O=C(CCC1CCCCC1)Nc1cccnc1
209	O=S(=O)(Nc1nc2c(s1)CCCC2)c1ccc(F)cc1	459	Cc1cc(OCC(O)CO)cc(C)c1Cl
210	O=C(Nc1ccccc1N1CCCCC1)c1ccc(Cl)cc1	460	CC1CC(=O)N(C)c2cc([N+](=O)[O-])ccc2N1
211	Cc1c[nH]c(CN(C)c2nc(C3CCCC3)nc3c2cnn3C)n1	461	CCCC(C)C(=O)Nc1ccc(F)c(Cl)c1
212	CCc1nc2ccccc2c(C(=O)Nc2ccc(F)cc2)c1C	462	Oc1c(Br)cc(F)c2cccnc12
213	Oc1nc(SCc2ccccc2Cl)nc2c1CCCC2	463	CCCCCc1nc2c(c(C)c1O)CCN2C
214	CC(O)c1ccc(c2ccc3ncnc(N4CCOCC4)c3c2)o1	464	CC(C)(C)NC(=O)OCCCc1c[nH]cn1
215	O=S(=O)(NC1C2CC3CC(C2)CC1C3)c1ccc(F)cc1	465	Cc1ccc(S(=O)(=O)N/N=C/C(C)C)cc1
216	CCc1ccc(C2COCCN2C(=O)Nc2cc(C)on2)o1	466	O=C(CC1CCCCC1)NCC1CCCO1
217	Cc1ccc(C)c(N2N=C(C(=O)Nc3cccnc3)CCC2=O)c1	467	O=C(CCn1ccnc1)Nc1cccc(O)c1
218	COc1ccc2c(=O)c(CCCCCC(F)(F)F)c(C)[nH]c2c1	468	COc1[nH]c(/N=C/c2ccco2)c(C#N)c1C#N
219	N#C/C(=C\c1ccc(F)c(Br)c1)C(=O)NC1CC1	469	O=[N+]([O-])c1ccc(N/N=C/c2ccc[nH]2)cc1
220	Cc1cccc(CNc2cc(c3ccccc3Cl)ncn2)c1	470	C/C1=C\CC[C@](C)(O)[C@H]2C[C@@H]2C(C)(C)C[C@H]1O
221	O=[N+]([O-])c1ccc(/C=N/Nc2nccc(C(F)(F)F)n2)cc1	471	O=C(Cc1cccs1)N/N=C/c1ccco1
222	C=S(=O)(NC(=O)c1ccc(C(F)(F)F)cc1)c1ccccc1	472	CCCNc1nc(C2CC2)nc(Cl)c1C
223	OC(C1=C/C(=C\c2ccncc2)c2ccccc21)c1ccncc1	473	COC1CCCCC1Nc1cc(C)ccc1C
224	C/C(=N\NC(=O)c1cccc(Br)c1)c1ccccn1	474	CC1(C)CC(=O)C2=CNc3ccccc3N=C2C1
225	N#Cc1ccc(c2[nH]c(c3ccccc3)nc2c2ccncc2)cc1	475	O=[N+]([O-])c1ccccc1C=NNc1ccccc1
226	Cc1ccn(c2nc(Nc3ccc(F)cc3)c3ncn(C)c3n2)n1	476	c1ccc(SCc2nc3ccccc3[nH]2)cc1
227	N#Cc1c(=O)c2nc(NCc3ccccc3)ncc2n2ccccc12	477	O=C(NCC(F)(F)C(F)F)c1cccnc1
228	CCc1cc(n2ccnc2)cnc1c1ccc(C2=NN=NN2)cc1	478	COc1ccccc1[S+]([O-])Nc1ccccc1
229	CC1=C(C(=O)Nc2ccccc2c2ccccc2)SCCO1	479	FC(F)(F)c1ccc2c(c1)N1CCCC1CN2
230	O=C(Nc1nc(c2ccccc2)nc2ccccc12)c1ccccc1	480	CCOc1ccccc1Nc1nnc(C)c(O)n1
231	O=C(NCc1ccc(F)cc1Cl)C1COc2ncccc21	481	CC(Cn1cccn1)NC(=O)c1ccsc1
232	O/N=C/c1cc(Cl)ccc1OCc1cccc2ccccc12	482	SC1=NC2(CCCCC2)Nc2ccccc21

**233**	COc1ccc(NCc2cc3cccc(C)c3nc2Cl)cc1	**483**	CC(C)(C)OC/C=C(/CO)[C@@H]1C=C[C@H](O)C1
**234**	CCCC(=O)CC[C@H]1OC(=O)c2c1cc(OC)c(OC)c2O	**484**	CCN(Cc1cnc[nH]1)c1ccc(C)c(C)c1
**235**	COc1cccc(Nc2ncc3c(n2)-c2ccccc2SC3)c1	**485**	Cn1ccc2c1C(=O)Nc1ccccc1S2
**236**	CSc1nc(Cl)c(C(F)(F)F)c(NC2CCCC2)n1	**486**	CC1=C[C@@]23CC(C)(C)C[C@@]2(O)[C@H](C)CC[C@@H]1O3
**237**	CC1=NN(c2ccccc2)C(=S)/C1=C/Nc1ccc(C)cc1C	**487**	c1ccc(CSc2nc3ccccc3[nH]2)cc1
**238**	Cn1ncc2c(N3CCCCC3)nc(Nc3ccccc3)nc21	**488**	COc1ccnc(NCc2cccnc2)c1C#N
**239**	Cc1sc2nc3ccc(NCc4ccco4)nn3c(=O)c2c1C	**489**	CC1(C)CC(=O)C=C(C#CC2(O)CCCCC2)C1
**240**	Cc1ccn2c(Nc3ccc(F)cc3)c(c3ccccn3)nc2c1	**490**	O=C(N/N=C/C=C/c1ccco1)c1ccncc1
**241**	CCOC(=O)c1noc2ncnc(Nc3ccc(F)cc3F)c12	**491**	Cc1cn2c(n1)CCC(NC(=O)CCC1CC1)C2
**242**	O=C(CSC1=NCCS1)Nc1nc2c(s1)CCCC2	**492**	C[C@@H]1CC[C@@H]2[C@H](C1)OC1(CCCC1)C[C@@]2(C)O
**243**	Cc1ccc2c(c1)CC/C2=N\Nc1nc(c2ccccc2)cs1	**493**	CCOC(=O)c1ccc(NC(=O)C2CC2)cc1
**244**	Cc1ccc2c(c1)c1c(n2C)CCCC1CNC(=O)C1CCC1	**494**	CCOCC(=O)Nc1ccc(C)c(Cl)c1
**245**	CCOC(=O)c1c2cc(O)ccc2oc1c1ccc(Cl)cc1	**495**	O=C(N/N=C/C=C/c1ccco1)c1cccnc1
**246**	Cc1nc(N[C@@H](C)C(C)(C)C)cc(c2ccc3ncsc3c2)n1	**496**	CCn1ncc(C(=O)Nc2cc(C)on2)c1C
**247**	Fc1cccc(CNc2nc(C(F)(F)F)nc3ccccc23)c1	**497**	CNc1cc(c2cnn(CC(C)C)c2)ncn1
**248**	CSc1ccc(c2cc(c3nc4ccccc4[nH]3)no2)cc1	**498**	Ic1cccc2[nH]ncc12
**249**	O=[N+]([O-])c1ccccc1Nc1nc2ccccc2n2cncc12	**499**	CCOC(=O)c1c[nH]n(c2ccc(C)cc2)c1=O
**250**	OCc1cnc2c(c1)c1ccccc1n2Cc1ccc(F)cc1F	**500**	Cc1sc2nc[nH]c(=[Se])c2c1C

REFERENCES

[1] N. C. Gilbert, J. Gerstmeier, E. E. Schexnaydre, F. Börner, U. Garscha, D. B. Neau, O. Werz, M. E. Newcomer, 'Structural and mechanistic insights into 5-lipoxygenase inhibition by natural products', *Nat Chem Biol* **2020**, *16*, 783-790 %787 20200511 %20200518 Jul %! Structural and mechanistic insights into 20200515-lipoxygenase inhibition by natural products %@ 20201552-20204469.

[2] M. J. Kobe, D. B. Neau, C. E. Mitchell, S. G. Bartlett, M. E. Newcomer, 'The structure of human 15-lipoxygenase-2 with a substrate mimic', *J Biol Chem* **2014**, *289*, 8562-8569.

[3] J. I. Mobbs, K. A. Black, M. Tran, W. A. C. Burger, H. Venugopal, T. R. Holman, M. Holinstat, D. M. Thal, A. Glukhova, 'Cryo-EM structures of human arachidonate 12S-lipoxygenase bound to endogenous and exogenous inhibitors', *Blood* **2023**, *142*, 1233-1242.

[4] D. Eisenberg, R. Luthy, J. U. Bowie, 'VERIFY3D: assessment of protein models with three-dimensional profiles', *Methods Enzymol* **1997**, *277*, 396-404.

[5] R. A. Laskowski, M. W. MacArthur, D. S. Moss, J. M. Thornton, 'PROCHECK: a program to check the stereochemical quality of protein structures', *Journal of Applied Crystallography* **1993**, *26*, 283-291.

[6] K. J. Bowers, E. Chow, H. Xu, R. O. Dror, M. P. Eastwood, B. A. Gregersen, J. L. Klepeis, I. Kolossvary, M. A. Moraes, F. D. Sacerdoti, J. K. Salmon, Y. Shan, D. E. Shaw, in *Proceedings of the 2006 ACM/IEEE conference on Supercomputing*, Association for Computing Machinery, Tampa, Florida, 2006, pp. 84–es.
